# Transcriptional and Chemical Changes in Soybean Leaves in Response to Long-Term Aphid Colonization

**DOI:** 10.3389/fpls.2019.00310

**Published:** 2019-03-13

**Authors:** Jessica D. Hohenstein, Matthew E. Studham, Adam Klein, Nik Kovinich, Kia Barry, Young-Jin Lee, Gustavo C. MacIntosh

**Affiliations:** ^1^Genetics and Genomics Graduate Program, Iowa State University, Ames, IA, United States; ^2^Bioinformatics and Computational Biology Graduate Program, Iowa State University, Ames, IA, United States; ^3^Ames Laboratory, United States Department of Energy, Department of Chemistry, Iowa State University, Ames, IA, United States; ^4^Division of Plant and Soil Sciences, Davis College of Agriculture, Natural Resources and Design, West Virginia University, Morgantown, WV, United States; ^5^Roy J. Carver Department of Biochemistry, Biophysics, and Molecular Biology, Iowa State University, Ames, IA, United States

**Keywords:** plant–insect interaction, soybean defense, isoflavones, feeding deterrence, *Aphis glycines*

## Abstract

Soybean aphids (*Aphis glycines* Matsumura) are specialized insects that feed on soybean (*Glycine max*) phloem sap. Transcriptome analyses have shown that resistant soybean plants mount a fast response that limits aphid feeding and population growth. Conversely, defense responses in susceptible plants are slower and it is hypothesized that aphids block effective defenses in the compatible interaction. Unlike other pests, aphids can colonize plants for long periods of time; yet the effect on the plant transcriptome after long-term aphid feeding has not been analyzed for any plant–aphid interaction. We analyzed the susceptible and resistant (*Rag1*) transcriptome response to aphid feeding in soybean plants colonized by aphids (biotype 1) for 21 days. We found a reduced resistant response and a low level of aphid growth on *Rag1* plants, while susceptible plants showed a strong response consistent with pattern-triggered immunity. GO-term analyses identified chitin regulation as one of the most overrepresented classes of genes, suggesting that chitin could be one of the hemipteran-associated molecular pattern that triggers this defense response. Transcriptome analyses also indicated the phenylpropanoid pathway, specifically isoflavonoid biosynthesis, was induced in susceptible plants in response to long-term aphid feeding. Metabolite analyses corroborated this finding. Aphid-treated susceptible plants accumulated daidzein, formononetin, and genistein, although glyceollins were present at low levels in these plants. Choice experiments indicated that daidzein may have a deterrent effect on aphid feeding. Mass spectrometry imaging showed these isoflavones accumulate likely in the mesophyll cells or epidermis and are absent from the vasculature, suggesting that isoflavones are part of a non-phloem defense response that can reduce aphid feeding. While it is likely that aphid can initially block defense responses in compatible interactions, it appears that susceptible soybean plants can eventually mount an effective defense in response to long-term soybean aphid colonization.

## Introduction

Aphids are hemipteran insects with specialized mouth parts, stylets, that facilitate efficient phloem sap feeding. In general, these insects alternate between parthenogenetic and sexual reproduction and can rapidly colonize their host plants. Due to their proficient colonization and settlement, combined with their ability to extract large amounts of photoassimilates from the plant and their role as vectors of plant viruses, aphids have a significant impact on cultivated plants throughout the globe (Goggin, 2007; Giordanengo et al., 2010). Most aphids are highly selective and feed only on one or a few plant species, although aphids that can feed on a large number of different plant species also exist. Non-host resistance, including physical barriers, nutritional quality, and deterrent secondary metabolites, determines whether aphids will feed on a particular plant. Aphids adapted to circumvent these barriers are able to feed on the host plant, where they trigger plant defenses based on the host plant immune system. Induced post-invasive mechanisms of resistance include pattern-triggered immunity (PTI) and effector-triggered immunity (ETI) (Kaloshian and Walling, 2016; Züst and Agrawal, 2016).

Upon contact with the invader, plant membrane receptors recognize highly conserved molecules associated with the surface of the pathogen or pest. In the case of pathogens, these microbe-associated molecular patterns (MAMPs) include chitin fragments and β-glucan fragments, cell wall and flagellum-derived peptides, and even intracellular proteins such as bacterial elongation factors, which are recognized by pattern recognition receptors (PRRs) initiating a signal transduction cascade that triggers plant defenses. The mechanisms used by plants to perceive herbivore attacks are similar to the perception of pathogens, and the resulting responses also share common themes. Herbivore-associated molecular patterns (HAMPs) are less characterized but it is clear that, at least for insects, salivary secretions play an important role in this recognition (Mithöfer and Boland, 2008; Hogenhout and Bos, 2011; Kaloshian and Walling, 2016). On the other hand, aphid salivary effectors can suppress host defenses to facilitate colonization, and it is likely that other aphid-induced changes such as the formation of galls and changes in the allocation of nutrients in the host are also mediated by salivary effectors (Hogenhout and Bos, 2011; Rodriguez and Bos, 2012; Kaloshian and Walling, 2016).

Induced plant responses triggered by pathogens and pests are mediated by blends of phytohormones that determine the intensity and composition of the response to each individual attacker. Hormones normally associated with defense include jasmonic acid (JA), ethylene (ET), and salicylic acid (SA), yet other phytohormones such as abscisic acid (ABA), cytokinins, gibberellins, and auxin also play roles in modulating plant defense responses (De Vos et al., 2005; Smith and Boyko, 2007; Asselbergh et al., 2008; Pieterse et al., 2009; Robert-Seilaniantz et al., 2011). Several plant–aphid interactions studies identified differential regulation of SA- and/or JA-mediated signaling pathways in response to aphid feeding. Aphids induce SA-regulated responses in Arabidopsis, *Nicotiana attenuata*, *Medicago truncatula*, wheat, tomato, sorghum, and barley. However, there does not seem to be a consensus on its overall impact on phloem-feeding insects (Thompson and Goggin, 2006; Goggin, 2007; Bari and Jones, 2009; Giordanengo et al., 2010; Morkunas et al., 2011; Kamphuis et al., 2013; Jaouannet et al., 2014). On the other hand, several studies have found strong evidence supporting an effective defense role of JA-mediated responses against several aphid species, even though JA-regulated markers are suppressed or only modestly induced in compatible aphid–plant interactions (Thompson and Goggin, 2006; Goggin, 2007; Howe and Jander, 2008; Bari and Jones, 2009; Giordanengo et al., 2010; Kamphuis et al., 2013; Jaouannet et al., 2014). Defense signaling pathways often lead to the production of compounds that can act as toxins against colonizing aphids. Many different plant secondary metabolites, including cardiac glycosides, alkaloids, benzoxazinoids, and glucosinolates, accumulate in response to aphid feeding in a variety of plant species. However, the ability of these compounds to reduce aphid performance depends on each specific aphid–plant interaction (Züst and Agrawal, 2016).

The soybean aphid (*Aphis glycines* Matsumura) causes significant yield loss and quality decline in soybean (*Glycine max*) (Ragsdale et al., 2007; Hesler et al., 2013). In addition to withdrawing photosynthates, soybean aphids alter the amino acid profile of the host plant (Chiozza et al., 2010), can vector soybean viruses (Hill et al., 2001), and excrete sugar-rich honeydew that promotes sooty mold fungal growth on leaves, which can interfere with photosynthesis. Due to these factors, uncontrolled aphid populations on susceptible plants can cause yield losses of up to 40% (Ragsdale et al., 2007). Several sources of host plant resistance have been identified in different plant introduction accessions and soybean cultivars, and nine have been genetically characterized (Hesler et al., 2013; Zhang et al., 2017). Among the resistance genes that have been fine-mapped to small regions of the soybean genome, *Rag1* is the best described. *Rag1* is a dominant gene that provides antibiosis and antixenosis against the soybean aphid (Hill et al., 2006a,b; Kim et al., 2010) and it has been mapped to a small region in soybean chromosome 7 that contains two NBS-LRR genes proposed as candidates to encode this resistance (Kim et al., 2010).

Transcriptome analyses showed that resistant plants carrying the *Rag1* gene mount a stronger and faster defense response to aphid feeding than susceptible plants. Li et al. (2007) compared the response of the susceptible Williams 82 soybean cultivar and the resistant (*Rag1*) Dowling cultivar at 6 and 12 h after aphid feeding and found a distinct resistance response with almost no overlap with the susceptible response. The number of differentially expressed (DE) genes in the resistant plant at 6 h was approximately twofold higher than the DE genes in the susceptible response at that time. The resistance response included genes related to cell wall metabolism, the phenylpropanoid pathway, and activation of the SA and JA signaling pathways. Following individual genes, these authors observed that the resistance response declines after 24 h (Li et al., 2007). A complementary analysis (Studham and MacIntosh, 2013) compared the response of resistant (*Rag1*) and susceptible near-isogenic soybean lines at 1 and 7 days after aphid colonization. In this case, a small resistance response was observed, but susceptible plants showed a significant number of DE genes at 1 day post-feeding that increased even more after 7 days. An analysis of phytohormone signals indicated that, at day 1, ET, JA, and SA biosynthesis and response genes were upregulated. However, at day 7, a reduced SA and no ET or JA response was observed despite the strong increase in expression of biosynthetic genes, particularly for JA. In parallel, a strong increase in ABA biosynthetic and response genes was observed at the late time point. These findings led to the hypothesis that, in compatible interactions, soybean aphids may induce a decoy response mediated by ABA that antagonizes effective JA, ET, and SA responses (Studham and MacIntosh, 2013).

An important and understudied aphid characteristic is the long association with their host plant. Unlike most other herbivores, aphids colonize plants for several weeks, yet very few studies have analyzed the effects of this long-term exposure on host physiology, gene expression, or metabolism. In this study we characterized the transcriptional response of soybean plants to long-term (21 days) soybean aphid infestation, using aphid-resistant (*Rag1*) and aphid-susceptible cultivars in a no-choice growth chamber experiment. We found a significant susceptible response that included induction of the isoflavonoid biosynthesis pathway and evidence of a general response to chitin in susceptible plants. On the other hand the resistance response was negligible, although we observed constitutive gene expression differences between the two soybean lines. Metabolite analyses corroborated accumulation of isoflavones in response to long-term aphid feeding on susceptible plants. Accumulation of these compounds outside of the plant vasculature suggested a non-phloem defense response. Choice experiments indicated that daidzein, one of the most highly induced isoflavonoids, is an aphid deterrent. Our results indicate that in contrast to plants exposed to aphids for 7 days, long-term exposure results in the induction of soybean defense responses.

## Materials and Methods

### Plant Growth Conditions and Aphid Infestations

Soybean (*Glycine max*) plants were grown in a growth chamber with a constant temperature of 25°C and a photoperiod of 16 h of light. The lights were a combination of incandescent and fluorescent bulbs, with an average light intensity of 375 μmol m^-2^ s^-1^ at the top of the plants. Plants were watered manually. Seeds were sown in SB300 Universal bark-based growing mix (Sun Gro Horticulture, Vancouver, BC, Canada) in 15 cm diameter green plastic pots. A dash of Rhizobium powder (*B.* *japonicum*) was applied to each seed. Aphids (*A. glycines* Matsumura) were obtained from a laboratory colony maintained in growth chamber with similar conditions to the experimental plants. The colony was kept on susceptible soybean plants. Thirty wingless, mixed age soybean aphids were applied to each plant on the V3 leaf, in the same manner as our previous study (Studham and MacIntosh, 2013). Twenty-four hours prior to sampling the aphids were counted on all plants. Two-tailed Student’s *t*-test was used to determine statistical differences in the number of aphids between genotypes (*p* ≤ 0.05).

### Experimental Design

This was a full-factorial no-choice experiment with two factors: soybean variety and aphid treatment. There were six plants per treatment. The two soybean varieties are aphid-resistant LD16060 (R) with the *Rag1* gene and aphid-susceptible SD01-76R (S). The aphid treatments were “with” (aphid plants) or “without” (control plants). The plant locations in the growth chamber were based on a split-split-plot randomized complete block design. The whole-plot factor was aphid treatment. After aphid infestation, plants were individually covered with nets (5-gal. paint strainers) (Trimaco LLC, Durham, NC, United States) secured with rubber bands around the pot. To minimize aphid contamination of control plants, all the aphid plants were on the left half of the chamber and the control plants (without aphids) were on the right. The split-plot factor was proximity to the back wall of the chamber. In previous experiments the plants closer to the back wall grew faster than the plants far from the back wall. Within each plot there were six complete blocks, and plants were randomized within each block.

### Leaf Sampling, RNA Isolation, and Microarray Analysis

Third trifoliate leaves were sampled after 21 days of aphid infestation. Each sample consisted of the third trifoliate leaves pooled from two plants. Aphids were removed from the leaf by first submerging the leaf in water and then gently rubbing off the aphids. Control plants were treated in the same manner to simulate aphid removal. Samples were frozen in liquid nitrogen, and then ground using a mortar and pestle. Total RNA was isolated, quality-checked, and quantified. GeneChip^®^Soybean Genome Arrays (Affymetrix, Santa Clara, CA, United States) were used to determine mRNA abundance in each of the samples, as described by Studham and MacIntosh (2013). The Affymetrix’s GeneChip Soybean Genome Array contains 37,600+ soybean probe sets that, according to SoyBase (Grant et al., 2010), correspond to an estimated 22,763 soybean genes, about 40% of the soybean genome (Schmutz et al., 2010). Triplicate samples were used for microarray analysis.

### Analysis of Microarray Data

The statistical analysis of the microarray data involved normalization and hypothesis testing of individual genes and gene sets. Raw intensities were normalized using the GCRMA method (Irizarry et al., 2003; Wu et al., 2004). A statistical model, which included aphid effects, genotypic effects, and the aphid:genotype interaction effect was created for each transcript, as described in detail in Studham and MacIntosh (2013). Differential expression for individual genes was determined using the following cutoffs: *p* ≤ 0.0001 and *q* ≤ 0.04 (FDR = 4%) and the absolute value of the fold change ≥ 2. For gene sets, the false discovery rate was 5%. Array probe sets were assigned to genes in the soybean genome (Soybean Genome Project, DoE Joint Genome Institute) by using BLASTN (Altschul et al., 1990; McGinnis and Madden, 2004) to compare probe set target sequences to predicted cDNA sequences. Only probe sets with sequences that matched a single gene with ≥ 95% identity and a resulting e-value ≤ 10^-30^ were assigned. DE genes were defined as soybean genes that have at least one matching DE probe set. The genes were annotated mainly by using the SoyBase and the Soybean Breeder’s Toolbox SoyChip Annotations (Hesler and Dashiell, 2007). GeneChip^®^Soybean Genome Array probe set annotations (Affymetrix) were also used in conjunction with the SoyBase annotations. Genes associated with the hormones abscisic acid (ABA), ethylene (ET), jasmonic acid (JA), and salicylic acid (SA) were used to analyze responses to long-term aphid infestation as described in Studham and MacIntosh (2012). The pathway score is based on fold change and significance of all genes associated with a hormone’s pathway. Raw and GCRMA-normalized datasets have been deposited in the NCBI’s Gene Expression Omnibus (Edgar et al., 2002) and are accessible through GEO Series accession number GSE115790^1^.

### Quantitative Real-Time Reverse-Transcribed PCR

An experiment identical to the one used for microarray analysis was carried out for confirmation of microarray results, using quantitative real-time reverse-transcribed PCR (qPCR) as described in Studham and MacIntosh (2013). Thus, the plant material used for confirmation was obtained independently of the material used for transcriptome analysis. The Pfaffl method (Pfaffl, 2001) was used to determine the fold change differences in transcript expression levels for each comparison. The efficiency for each gene was determined using a standard curve based on the dilution series. Results were normalized using the reference gene ubiquitin (*Glyma.20g141600*), which is unaffected by aphid treatment and genotypic differences based on the microarray data presented here and data obtained in Studham and MacIntosh (2013). Statistical analysis was performed using *t*-test (*p* < 0.05).

### Phenylpropanoid Pathway Analysis

Our representation of the soybean phenylpropanoid pathway was elucidated based on literature (Schopfer et al., 1998; Jung et al., 2000; Akashi et al., 2005, 2009; Bomati et al., 2005; Kim et al., 2005; Ralston et al., 2005; Yu and McGonigle, 2005; Deavours et al., 2006; Suzuki et al., 2006; Zabala et al., 2006; Nagamatsu et al., 2007; Tuteja and Vodkin, 2008; Fliegmann et al., 2010; Yi et al., 2010), the MedicCyc *Medicago truncatula* database (Urbanczyk-Wochniak and Sumner, 2007; Mensah et al., 2008), and the MetaCyc Encyclopedia of Metabolic Pathways (Kim et al., 2008; Caspi et al., 2009). In the diagram, the susceptible response microarray result for each gene is represented by a circle near the reaction line for the enzyme it encodes. The size of the circle is proportional to the absolute value of the fold change, the color hue indicates the direction of the change (yellow for up, blue for down), and the color saturation is proportional to the statistical significance of the change. If a gene’s results failed to meet relaxed differential-expression criteria (| fold|≥ 2, *p* ≤ 0.05) then it is unchanged and shown as a small gray circle. Normally a gene’s results are based on one probe set, but in cases in which a gene has multiple Affymetrix probe sets assigned, the probe set with the lowest *p*-value is chosen to represent the gene as long as all the probe set changes are in the same direction or unchanged. If a gene’s probe sets changed in both directions then the gene is shown to be unchanged.

### Metabolite Quantification and Mass Spectrometry Imaging

An experimental design similar to the microarray experiment was used to determine isoflavone accumulation in response to aphid feeding. Third trifoliates from control and aphid-infested plants were collected 21 days after infestation.

One leaflet from each trifoliate was used for metabolite quantification. Leaflets were freeze-dried, ground using mortar and pestle in liquid N_2_, and extracted on a platform shaker at 150 rpm for 16 h with 80% ethanol (25 μl mg^-1^). Cyanidin-3-*O*-glucoside (50 μM) was used as an external standard. Where indicated, ethanolic extracts were hydrolyzed with 1N HCl at 90°C for 2 h. The solvent was evaporated under vacuum, and the aqueous phase extracted twice with an equal volume of ethyl acetate and the organic phase evaporated to dryness. The residue was resuspended in 80% ethanol, filtered through 0.2 μm PVDF, and 15 μl aliquots were injected onto a Symmetry C18 column (Waters Corporation, 4.6 mm × 75 mm, 100 Å, 3.5 μm) held at 30°C, and analyzed at 254 nm using a Waters Alliance 2695 HPLC equipped with PDA. The mobile phase flow rate was 1 mL min^-1^ and consisted of buffers A [0.1% (v/v) formic acid in water] and B [0.1% (v/v) formic acid in acetonitrile], with the following gradient (0 min 95% A, 3 min 85% A, 4 min 85% A, 21 min 50% A, 21.1 min 20% A, 24 min 20% A, 24.1 min 95% A, 28 min 95% A) using a linear gradient between time points. Compounds were quantified by comparison to standard curve of authentic standards. Daidzein and formononetin (Indofine, Inc.) were a generous gift from Dr. Brian McGonicle (DuPont). Genistein and kaempferol were purchased from Extrasynthese (France). Glyceollins were analyzed by UPLC-PAD-MS as described in Farrell et al. (2017).

A second leaflet from each trifoliate was used for mass spectrometry imaging (MSI). Leaflets were imprinted on a polytetrafluoroethylene (PTFE) membrane and analyzed using MALDI-MSI with 1,5-diaminonaphthalene (DAN) as a matrix in negative ion mode, following the method previously described by Klein et al. (2015). Relative quantification was carried out by combining ion signals for each analyte over the tissue area, then normalized to the ion signal for DAN.

In both experiments, statistically significant differences were determined by two-tailed Student’s *t*-test (*p* < 0.05).

### Aphid Choice Experiment

First trifoliates were excised from susceptible plants at the V2–V3 stage and grown in a growth chamber under conditions similar to those used for microarray analysis. Petioles of individual leaves were placed in 2 ml tubes containing control solution (0.6–1.6% DMSO in water) or isoflavone solution (isoflavone in DSMO dissolved in water). One control and one isoflavone treated trifoliate were then paired and connected with a piece of filter paper between leaves. After 6 h, 10 adult aphids were placed on the filter paper and allowed to move freely between the two trifoliates. The number of aphids feeding on each leaf was recorded after 16 h. Only adult aphids were counted. Three independent experiments with at least 10 leaf pairs were carried out. Statistically significant differences were determined by paired Student’s *t*-test (*p* < 0.05).

## Results

### Identification of Potential Defense Responses to Long-Term Aphid Colonization Through Transcriptome Analysis

**FIGURE 1:**
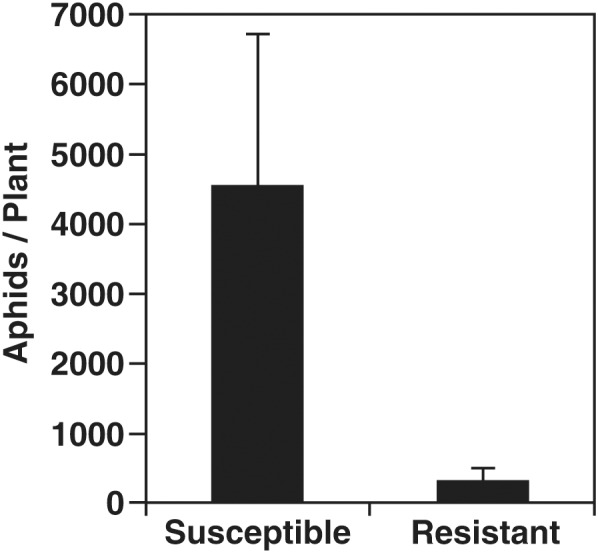
Aphid population levels after 20 days of infestation. Susceptible (SD01-76R) and aphid-resistant (LD16060) soybean plants were infested with 30 aphids and aphid populations were quantified after 20 days. The difference in aphid number between resistant and susceptible plants was statistically significant (*p* ≤ 0.05; two-tailed Student’s *t*-test). Error bars represent standard error of the mean (*n* = 18).

A no-choice growth chamber experiment was conducted using two related cultivars: aphid-susceptible SD01-76R (S plants), and aphid-resistant LD16060 (R plants) carrying the *Rag1* aphid resistance gene. At the V3 growth stage 50% of the plants were infested with soybean aphids of biotype 1 which is controlled by *Rag1*. After 20 days of infestation, aphids on each plant were counted, and after 21 days of infestation the leaves were harvested to undergo mRNA profiling. The aphid population increased in R and S varieties, but S plants had significantly higher aphid counts, about 14-fold, than R plants, confirming the resistance phenotype of LD16060 (Figure 1). For a more detailed description of aphid population growth on these soybean lines, see Chiozza et al. (2010). Although heavy infestations may result in plant stunting and leaf yellowing (Tilmon et al., 2011), the leaves used in our analysis did not show strong symptoms besides deposition of honeydew and some decrease in the intensity of leaf color.

**FIGURE 2:**
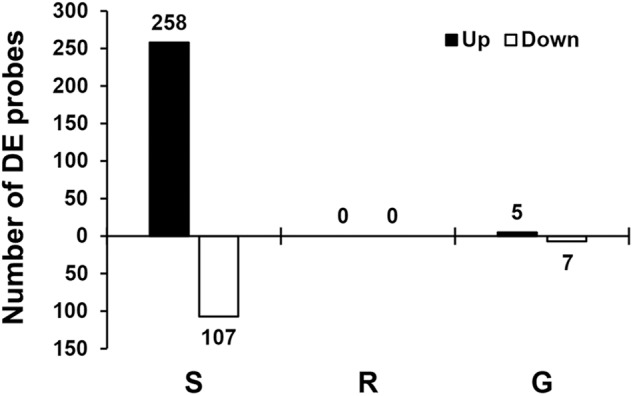
Transcriptional responses to aphid infestation and genetic differences. Numbers of DE soybean probes for three different comparisons: susceptible response (S) comparing gene expression in susceptible plants with and without aphids, resistant response (R) comparing gene expression in resistant plants with and without aphids, and genetic differences (G) comparing gene expression in resistant and susceptible plants without aphids. Differential expression for individual probes was determined using the following cutoffs: *p* ≤ 0.0001 and *q* ≤ 0.04 (FDR = 4%) and the absolute value of the fold change ≥ 2. The number of DE probes is indicated above or under each bar.

Transcript levels were determined using Affymetrix’s GeneChip Soybean Genome Array. We focused on three comparisons (Supplementary Table 1): the susceptible response (infested S plants vs. uninfested S plants), the resistance response (infested R plants vs. uninfested R plants), and genetic differences (uninfested R plants vs. uninfested S plants). Although the comparison between infested S and R plants is also reported (Supplementary Table 1), this result was not considered a reliable comparison given the large difference in number of aphids infesting each genotype and thus is not discussed here. Transcripts corresponding to each probe set were considered to be DE if they had a statistically significant (*p* ≤ 0.0001 and *q* ≤ 0.04) change of at least twofold. Overall our cutoffs were very conservative and our false discovery rate was lower than 4% (Storey et al., 2004). Figure 2 shows the number of probe sets with DE transcripts per comparison. The susceptible response consisted of 365 probe sets mapped to 252 soybean genes according to the current genome annotation (Glyma version 2.0), the resistant response did not return any DE transcript with the cutoffs set for our experiment, and the genetic differences consisted of 12 probe sets mapped to 10 soybean genes.

#### Susceptible Response

The susceptible response was the only substantial transcriptional change observed for the three comparisons in this experiment, and it was predominantly associated with defense processes. Overall 258 probes (182 genes) were induced and 107 probes (70 genes) were suppressed. Supplementary Table 1 lists all DE probes and corresponding genes for this comparison.

In order to identify biological processes associated with the S response, we determined the overrepresentation of Gene Ontology (GO) terms associated with probe sets in our DE list with respect to the full array (Morales et al., 2013). The molecular processes and biological functions overrepresented in our dataset and the DE genes included in each category are shown in Supplementary Table 2. The most significantly overrepresented gene set corresponded to genes involved in the response to chitin, with 49 DE genes in the susceptible response. Other defense-related processes included response to insect, response to wounding, regulation of defense response, response to fungus, and innate immune response. Signaling processes associated with defense responses, such as MAPK cascade, respiratory burst involved in defense response, regulation of hydrogen peroxide metabolic process, detection of biotic stimulus, regulation of plant-type hypersensitive response, were also overrepresented in the S response.

Several phytohormone-associated responses were also overrepresented, including ethylene biosynthetic process, salicylic acid-mediated signaling, salicylic acid biosynthetic process, and jasmonic acid-mediated signaling pathway. Upregulation of JA and SA signaling was observed in the S response to aphid colonization in soybean. A comparable result was obtained when we analyzed the phytohormone response using a previously described bioinformatics tool (not shown) (Studham and MacIntosh, 2012). Remarkably, abscisic acid signaling was absent, in contrast to the strong ABA response observed in the susceptible response after 7 days of aphid feeding (Studham and MacIntosh, 2013).

We also carried out a different gene set analysis, using the GSA method (Efron and Tibshirani, 2007) that examines the full dataset instead of just the DE genes for each response, and calculates differentially regulated gene sets. This analysis returned similar gene sets associated with defense responses and phytohormone pathways for the S response (Supplementary Table 3).

Analysis of individual DE genes revealed a large proportion of transcription factors (36 out of 252 genes, 14.3%) in the S response. Most of these genes were induced by aphid infestation and they correspond to several transcription factor families (Table 1). The two families with larger representation correspond to the WRKY (11 upregulated genes) and NAC (6 upregulated genes) families, which are normally associated with defense responses (Nuruzzaman et al., 2013; Bakshi and Oelmüller, 2014).

**Table 1:** Differentially expressed genes encoding transcription factors in the susceptible response.

Gene ID	Class	Fold change	Top Arabidopsis (TAIR10) BLASTP Hit
*Glyma.16G199000*	AP2	42.78	AT5G51990, DEHYDRATION-RESPONSIVE ELEMENT-BINDING PROTEIN 1D, DREB1D
*Glyma.09g147200*	AP2	32.86	AT5G51990, DEHYDRATION-RESPONSIVE ELEMENT-BINDING PROTEIN 1D, DREB1D
*Glyma.02G132500*	AP2	16.66	AT4G34410, redox responsive transcription factor 1, RRTF1
*Glyma.02G006200*	AP2	4.84	AT3G23240, ethylene response factor 1, ERF1
*Glyma.10G204400*	AP2	4.02	AT1G25560, ethylene response DNA binding factor 1, EDF1
*Glyma.09G247000*	bHLH	–2.78	AT4G37850, basic helix-loop-helix (bHLH) DNA-binding superfamily protein
*Glyma.03G173300*	C2H2 and C2HC	27.73	AT2G37430, ZAT11
*Glyma.10G045400*	Zinc fingers	11.38	AT3G53600, C2H2-type zinc finger family protein
*Glyma.15G040700*		10.86	AT2G28710, C2H2-type zinc finger family protein
*Glyma.19G174200*		5.23	AT2G37430, ZAT11
*Glyma.04G008900*	GATA zinc finger	6.81	AT4G36240, GATA transcription factor 7, GATA7
*Glyma.07G266500*	GRAS	9.74	AT4G17230, SCARECROW-like 13, SCL13
*Glyma.12G137700*	GRAS	5.37	AT5G48150, PAT1
*Glyma.13G285400*	GRAS	4.60	AT1G50600, SCARECROW-like 5, SCL5
*Glyma.03G221700*	MYB	5.80	AT2G47190, ATMYB2
*Glyma.20G209700*	MYB	5.26	AT3G23250, ATMYB15
*Glyma.11G182000*	NAC	7.83	AT5G22380, NAC domain containing protein 90, NAC090
*Glyma.04g226700*	NAC	7.26	AT4G35580, NAC transcription factor-like 9, NTL9
*Glyma.06G138100*	NAC	6.79	AT4G35580, NAC transcription factor-like 9, NTL9
*Glyma.11G096600*	NAC	5.72	AT2G17040, NAC domain containing protein 36, NAC036
*Glyma.16G016700*	NAC	3.46	AT4G35580, NAC transcription factor-like 9, NTL9
*Glyma.07G048100*	NAC	2.80	AT4G35580, NAC transcription factor-like 9, NTL9
*Glyma.12G183800*	Other	3.93	AT2G27580, A20/AN1-like zinc finger family protein
*Glyma.08G044400*	Other	3.08	AT1G73805, SAR DEFICIENT 1, SARD1
*Glyma.03G258400*	Other	–2.62	AT4G00950, maternal effect embryo arrest 47, MEE47
*Glyma.08G021900*	WRKY	13.73	AT4G11070, ATWRKY41
*Glyma.05G215900*	WRKY	9.61	AT4G11070, ATWRKY41
*Glyma.17G222300*	WRKY	9.20	AT1G80840, ATWRKY40
*Glyma.14G102900*	WRKY	8.12	AT1G80840, ATWRKY40
*Glyma.04g218700*	WRKY	6.45	AT5G64810, ATWRKY51
*Glyma.01G128100*	WRKY	5.89	AT2G38470, ATWRKY33
*Glyma.19G254800*	WRKY	5.68	AT4G11070, ATWRKY41
*Glyma.01G224800*	WRKY	5.46	AT4G11070, ATWRKY41
*Glyma.06g147100*	WRKY	4.67	AT5G64810, ATWRKY51
*Glyma.17G222500*	WRKY	3.95	AT1G80840, ATWRKY40
*Glyma.04G223300*	WRKY	3.45	AT3G56400, ATWRKY70


Since GO-term analysis indicated a preponderance of defense processes, many of which are associated with PTI, we searched our dataset for key components of this pathway. Among DE genes in the susceptible response we identified increased expression for key regulators of PTI (Bigeard et al., 2015), including *MPK3* (*Glyma.U021800*), orthologs of *AtWRKY33* (*Glyma.01G128100*) and *AtPTi1–4* (*Glyma.10G009100*), and several receptor-like kinases and resistance proteins. Four orthologs (*Glyma.03G201000, Glyma.03G201100, Glyma.03G201300*, and *Glyma.03G201500*) of *NHL10*, a common marker of the PTI pathway (Boudsocq et al., 2010), were also strongly induced in response to aphids. Finally, many components of the immune secretory pathway were also induced in response to aphid feeding. This is one of the last steps in PTI, and is responsible for callose deposition and potentially responsible for the release of phytoalexins and cell wall modifying activities (Yun et al., 2016; Klink et al., 2017). Genes related to cell wall modification and amino acid metabolism and transport, processes commonly associated with the response to aphid feeding were also identified in the susceptible response set.

**FIGURE 3:**
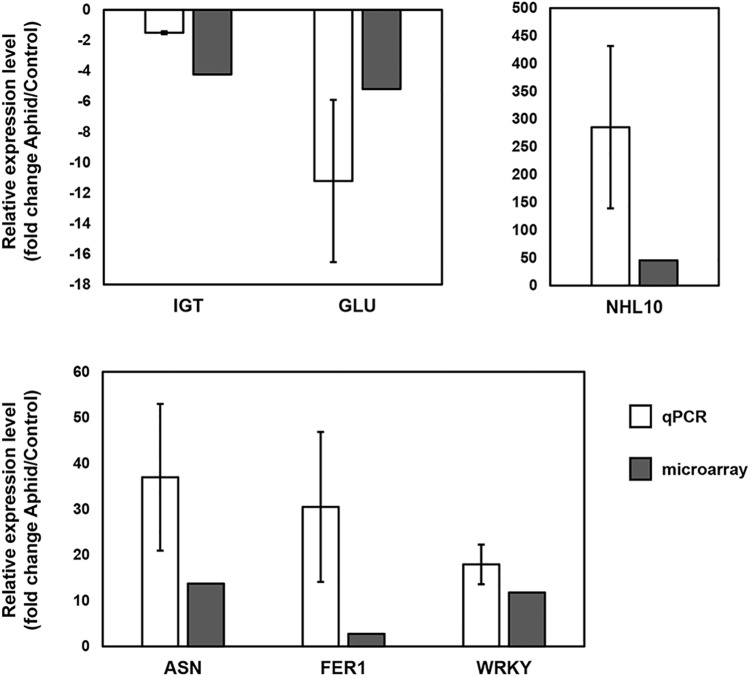
Confirmation of microarray results for selected transcripts. Quantitative real-time reverse-transcribed PCR (qPCR) was used to confirm the microarray results for changes seen in the susceptible response and genetic differences. Isoflavonoid glycosyltransferase (IGT, *Glyma.15g221300*) and endo-1,4-β-glucanase (GLU, *Glyma.08g022300*) were downregulated and asparagine synthetase 1 (ASN, *Glyma18g06840*), NHL10 (*Glyma.03g201300*) and a WRKY transcription factor (*Glyma.05G215900*) were upregulated in the susceptible response. Ferritin-1 (FER1, *Glyma.18G205800*) was expressed at higher levels in uninfested resistant plants than in uninfested susceptible plants. Fold change is indicated for each transcript as determined by qPCR and microarray analysis. All gene expression differences between infested and uninfested or between S and R plants were statistically significant (*p* < 0.05 for qPCR, *p* ≤ 0.0001 and *q* ≤ 0.04 for microarray). Error bars represent standard error of the mean (*n* = 3).

Quantitative RT-PCR of selected genes was used to confirm the microarray results. Figure 3 shows the results for *isoflavonoid glycosyltransferase* and *endo-1,4-β-glucanase* (repressed in response to aphid colonization), and *asparagine synthetase 1*, *WRKY41*, and *NHL10* (induced in response to aphid colonization), which correlate well with the results observed in our microarray analysis.

**FIGURE 4:**
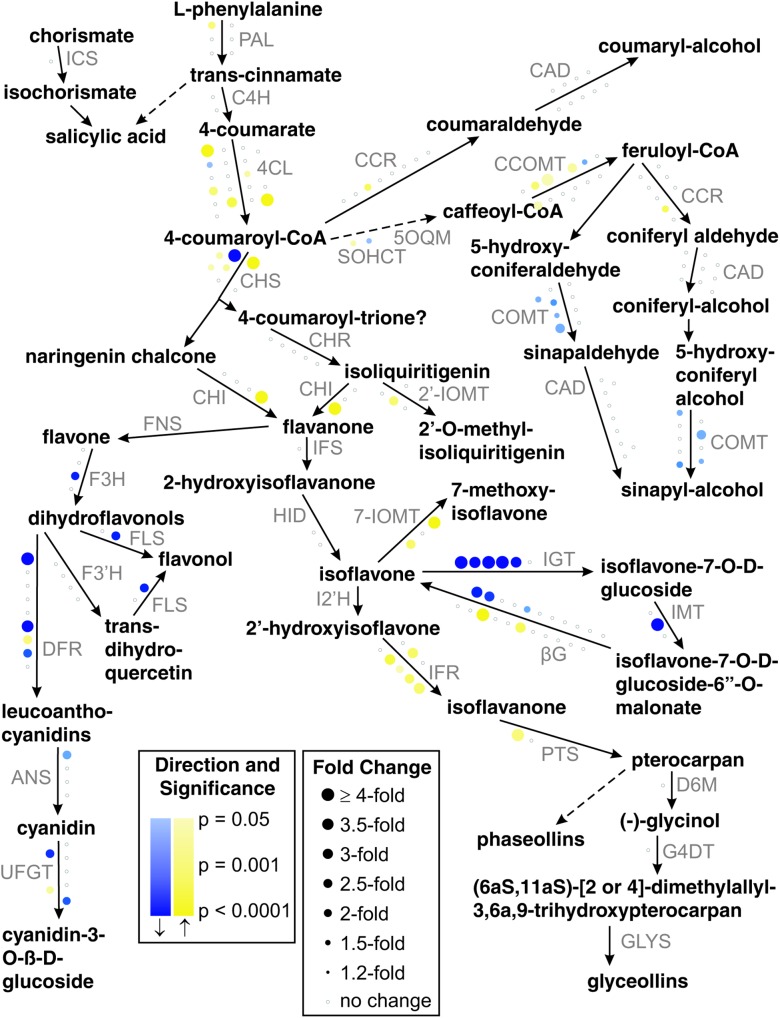
Changes in transcripts related to phenylpropanoid metabolism in response to aphid feeding in susceptible plants. A subset of the phenylpropanoid pathway is shown, with enzyme abbreviations in gray font. Each circle represents a soybean gene and its microarray result is depicted by its color hue, color saturation, and size. Genes whose results meet relaxed DE criteria (| fold| ≥ 2, *p* ≤ 0.05) are shown in blue (suppressed) or yellow (induced). If a gene’s results fail to meet the relaxed criteria then they are represented by a small white circle with a gray outline. Color intensity indicates statistical confidence and circle size indicates fold change.

One of the overrepresented gene sets in the GO term analysis is quercetin 3-*O*-glucosyltransferase activity, suggesting that flavonoids may participate in the response to aphids. A more detailed analysis of the phenylpropanoid pathway looking at individual DE genes indicated that this pathway was strongly affected by aphid infestation. We detected 15 DE genes, five upregulated and 10 downregulated (Table 2). To analyze the aphid-induced changes on the phenylpropanoid pathway in more detail, we performed a systematic analysis of phenylpropanoid metabolism using relaxed statistical significance and fold change criteria (absolute fold change ≥ 2, *p* ≤ 0.05). The pathway diagram in Figure 4 illustrates these results on a simplified sub-network of phenylpropanoid reactions. Out of 180 genes analyzed, 57 (31.7%) met the relaxed criteria and these genes represent transcripts encoding most of the enzymes shown in the simplified pathway. The flavonol and lignin branches of the pathway appear to be suppressed or unchanged, while transcripts corresponding to the defense-related isoflavonoid branch are mostly induced. Isoflavonoid glycosyltransferase, which converts the bioactive isoflavone aglycone into its glycoside conjugate, is strongly downregulated. Three of the six soybean genes that are annotated as flavonoid glycosyltransferases are significantly downregulated using strict criteria and five of the six genes are downregulated according to the relaxed criteria.

**Table 2:** Differentially expressed genes related to the phenylpropanoid pathway in the susceptible response.

Probe ID	Gene ID	Annotation	Microarray results		
			Fold change	*p*-Value	*q*-Value
Gma.7423.2.S1_a_at	*Glyma.11g198300*	Similar to 4-coumarate-CoA ligase	+14.16	1.09E-05	2.99E-03
GmaAffx.87547.2.S1_at	*Glyma.11g129600*	Glycoside hydrolase, family 1	+9.68	6.61E-07	7.16E-04
Gma.17605.3.S1_at	*Glyma.08g109500*	Chalcone synthase 1	+5.73	2.03E-05	3.91E-03
GmaAffx.36482.1.S1_at	*Glyma.13g302500*	Anthocyanin acyltransferase	+4.42	4.11E-05	5.54E-03
Gma.3260.1.S1_at	*Glyma.20g213700*	Caffeate *O*-methyltransferase	+3.74	4.66E-05	5.94E-03
GmaAffx.5737.1.S1_at	*Glyma.15g054300*	Isoflavonoid glycosyltransferase	–3.31	1.53E-05	3.47E-03
Gma.1527.2.S1_at	*Glyma.11g164700*	Dihydroflavonol reductase	–3.55	9.63E-05	9.22E-03
GmaAffx.49284.1.A1_s_at	*Glyma.16g033700*	UDP-glycose:flavonoid glycosyltransferase	–3.78	8.09E-05	8.31E-03
GmaAffx.70258.1.S1_s_at	*Glyma.02g048400*	Flavanone-3-hydroxylase	–3.94	1.47E-05	3.47E-03
GmaAffx.25369.1.S1_s_at	*Glyma.11g053400*	Isoflavonoid glycosyltransferase	–4.00	1.65E-05	3.53E-03
Gma.11753.1.S1_at	*Glyma.15g221300*	Isoflavonoid glycosyltransferase	–4.23	1.40E-05	3.38E-03
Gma.5757.1.S1_at	*Glyma.18g258000*	Isoflavonoid malonyl transferase 2	–4.61	2.92E-05	4.69E-03
Gma.9072.1.S1_at	*Glyma.19g105100*	Chalcone synthase	–4.97	4.29E-05	5.68E-03
Gma.1527.1.S1_x_at	*Glyma.18g057900*	Dihydroflavonol reductase	–5.22	4.14E-06	1.77E-03
Gma.15687.1.A1_at	*Glyma.02g104600*	UDP-glucuronosyl/UDP-glucosyltransferase	–5.66	8.81E-05	8.77E-03


#### Resistant Response

Expression of none of the 37,653 probe sets changed in response to long-term aphid infestation in the R plants, using our strict criteria. This is similar to results in our previous transcriptional profiling in resistant plants after 1 and 7 days of aphid infestation (Studham and MacIntosh, 2013). Despite the lack of individual DE genes, gene set analysis using the GSA method indicated that there were DE gene sets (Supplementary Table 3). Several of the gene sets induced by aphids were related to defense, including genes associated with JA and ET-dependent systemic resistance.

#### Genetic Differences

Although the resistant and susceptible cultivars used in this study are closely related (Chiozza et al., 2010), the uninfested R and S plants still have transcriptional differences (see Supplementary Table 1). Twelve probes, corresponding to 10 annotated genes, were DE in control plants. A Ribonuclease H transcript was present at high levels in S plants but it was nearly undetectable in R plants. S plants also had higher levels of transcripts corresponding to an isoflavone 4-*O*-methyltransferase, an amino acid decarboxylase and candidate resistance protein KR1. Two thioesterases and a phospholipase D gene were found at higher levels in R plants than S plants. The GO-term analysis using GSA (Supplementary Table 3) indicated that cell wall metabolism transcripts are more abundant in S plants, as was the general “defense response” set. Even though these plants never had aphids or any observable disease, defense-related gene sets and individual genes are DE in this comparison.

Previous transcriptome analyses had identified ferritins as potential components of soybean resistance and tolerance to aphids (Li et al., 2007; Prochaska et al., 2015). Our statistical model indicated that one of the effects of the resistant genotype was an increase in all ferritin transcripts except for *ferritin-3* (see Supplementary Table 4 for detailed ferritin results). Our statistical model reveals that not only are ferritins 1, 2, and 4 expressed at approximately twofold higher levels in R plants in the current experiment, but also in a previous experiment in which we analyzed plants at earlier stages (Studham and MacIntosh, 2013). These genes were among those most consistently upregulated by the genetic effect in the statistical model. The hypothesis testing resulted in modest fold change increases and low *p*-values, but the *q*-values were too high for these transcripts to meet our strict differential expression criteria. The differential expression of one of the genes, *ferritin-1* (*Glyma.18g205800*), was confirmed by qPCR (Figure 3). These results suggest that iron homeostasis may be important in resistance against aphids in soybean.

### Isoflavones May Be Part of the Defense Mechanisms Against Soybean Aphids

#### Isoflavones Accumulate in Response to Long-Term Aphid Colonization

**FIGURE 5:**
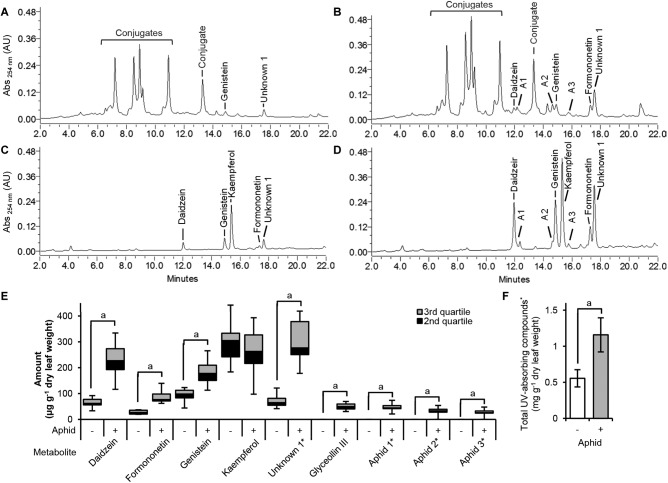
Metabolite changes in the susceptible response. HPLC-PAD chromatograms of non-hydrolyzed ethanolic extracts **(A,B)** and acid-hydrolyzed extracts **(C,D)** of untreated **(A,C)** and aphid-treated **(B,D)** susceptible soybean leaves. Measurable amounts of the isoflavonoid aglycones daidzein and formononetin were found only the aphid-treated extracts **(B)**, not in the untreated samples **(A)**. Hydrolyzable UV-absorbing compounds (Conjugates) were also increased in aphid-treated leaves **(A,B)**. Acid hydrolysis of extracts demonstrated that the major increases were derived from the isoflavonoids daidzein, genistein, and formononetin, in addition to an unknown compound (Unknown 1; λmax 237.9 nm, shoulder 285.2 nm), whereas kaempferol-derived compounds were unchanged **(E)**. ^∗^Unknown compounds were quantified based on daidzein equivalents. Aphid1–3 represents unknown compounds detected only from aphid-treated samples. Amounts of total UV-absorbing metabolites increased twofold in the aphid-treated extracts **(F)**. Error bars represent standard error of the mean (*n* = 7–9). A lower case **a** indicates significant differences (*p* < 0.05; two-tailed Student’s *t*-test).

Our transcriptome analysis identified many DE genes corresponding to the phenylpropanoid pathway in the susceptible response. Moreover, a detailed analysis of this pathway suggested that the isoflavonoid branch is upregulated while the lignin and flavonoid branches are unchanged or suppressed. To determine whether the changes in gene expression truly reflect changes in metabolite levels, flavonoids and isoflavonoids were extracted from control and aphid-infested leaves and then quantified by comparison to authentic standards using HPLC-PAD (Figure 5). Amounts of total UV-absorbing metabolites increased twofold after aphid feeding (Figure 5F). Only the aphid-treated samples accumulated measurable amounts of the isoflavonoid aglycones daidzein and formononetin (Figure 5A,B). However, their levels were low compared to other compounds that were found to be increased in the aphid-treated samples. To determine the identity of the other compounds, acid hydrolysis was performed. The major increases in aphid-treated samples were (iso)flavonoid-derived conjugates (Figure 5C,D). Quantification of individual peaks from the hydrolyzed samples showed significant increases in the isoflavones daidzein, genistein, and formononetin, and to a minor extent glyceollin III (Figure 5E). We also observed a significant increase for a non-identified metabolite (Unknown 1) that reached high levels in aphid-infested plants, as well as three minor peaks (Aphid 1–3) that were only found in aphid-treated samples. On the other hand, levels of the flavonol kaempferol were not affected by aphid colonization, confirming that while the isoflavonoid branch was induced, aphids had no effect on the flavonoid branch.

#### Isoflavones Are Part of a Non-phloem Defense Mechanism Against Soybean Aphids

**FIGURE 6:**
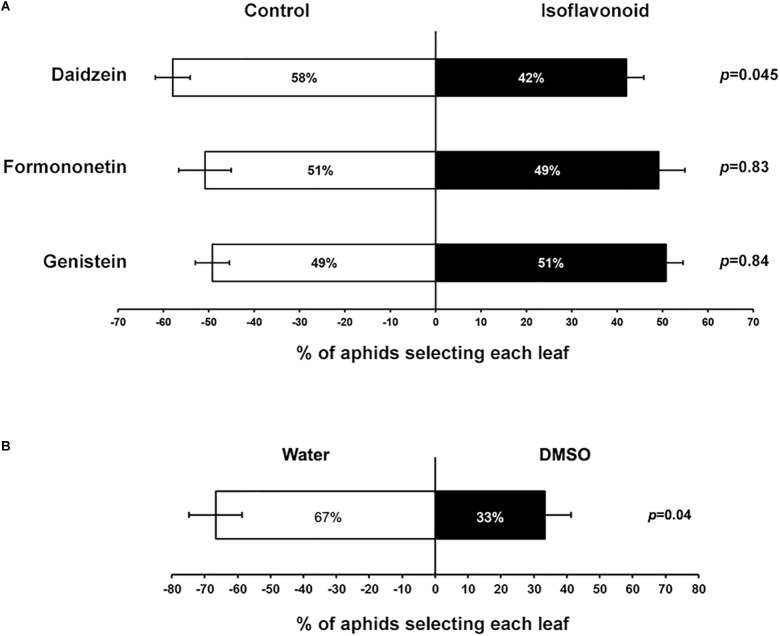
Aphids choose between control and daidzein-treated plants. **(A)** Petioles of individual leaves of susceptible plants were placed on tubes containing either DMSO (control) or different isoflavone solutions (5 μg ml^-1^). One control and one isoflavone-treated leaf were then paired and connected with a piece of filter paper between leaves. Ten adult aphids were placed on the filter paper and allowed to move freely between the two trifoliates. The number of aphids feeding on each leaf was recorded after 16 h. **(B)** Choice experiment as in **(A)**, using water vs. DMSO (0.6–1.2% v/v). Statistically significant differences were determined by paired Student’s *t*-test. Error bars represent standard error of the mean (*n* = 38–49).

Transcriptome changes and accumulation of several isoflavones in response to aphid feeding suggested that these metabolites could have an effect on aphid feeding or colonization ability and thus be part of an effective defense mechanism. Although an *in vitro* feeding protocol has previously been reported for soybean aphids (Wille and Hartman, 2008), in our hands very high mortality (even in control samples) precluded us from testing any isoflavonoid toxicity effect directly. However, we were able to develop a choice assay to test whether the presence of isoflavonoids affected aphid feeding preferences. For this analysis, soybean leaves were detached from the plant and their petioles placed in tubes with either a control solution containing DMSO used as solvent, or a solution containing 5 μg ml^-1^ of daidzein, formononetin, or genistein. After 8 h, leaves were arranged in pairs, including a control and an isoflavone-treated leaf, connected by a filter paper where 10 aphids were deposited. Aphids were able to move freely between the two leaves, and the location of aphids was recorded 16 h after aphids were released. In this choice test daidzein had a significant deterrent effect on soybean aphids, while formononetin and genistein had no effect at the concentration tested (Figure 6A). It is important to note that the DMSO used to prepare the stock isoflavonoid solutions also has a negative effect on aphid preference (Figure 6B). Thus, the strength of the deterrent effect observed for daidzein could be partially masked by the solvent.

**FIGURE 7:**
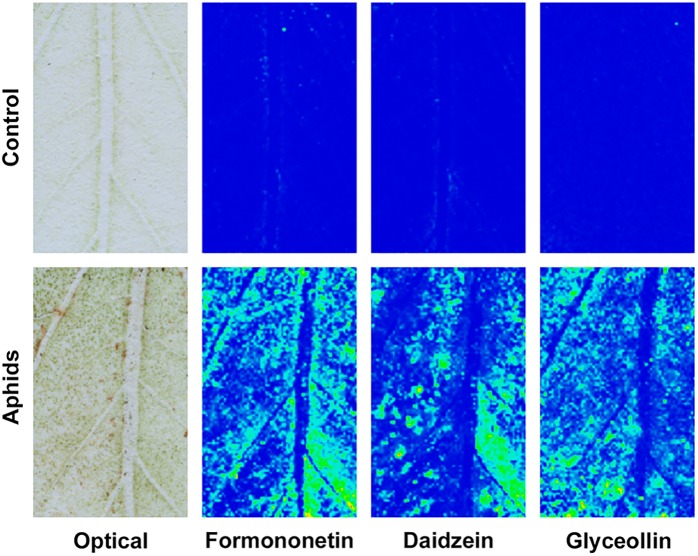
Isoflavonoids do not accumulate in leaf vasculature. Analysis of the distribution of isoflavonoids in susceptible control leaves and susceptible leaves colonized by aphids for 21 days was carried out using negative mode MALDI-MSI after imprinting leaves on a PTFE surface. Left panels, optical image of imprinted PTFE. Other panels, chemical images for formononetin (*m*/*z* 267.066), daidzein (*m*/*z* 253.05), and glyceollin (*m*/*z* 337.108) using an arbitrary scale (blue = low; yellow = high). Representative images are shown.

**FIGURE 8:**
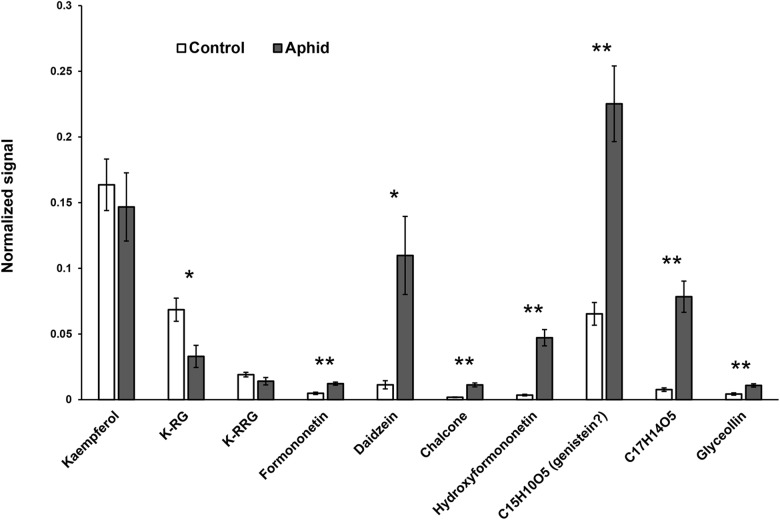
Determination of isoflavone accumulation in response to aphid feeding through mass spectrometry imaging (MSI). Relative quantification was carried out by acquisition of total signal for individual ions in a fixed rectangle placed randomly over each image, then normalized to the ion signal for 1,5-diaminonaphthalene (matrix). *m*/*z* for each compound: kaempferol, 285.04; kaempferol-3-rhamnoglucoside (K-RG), 593-152; clitorin (K-RGG), 739.211; formononetin, 267.066; daidzein, 253.05; chalcone, 255.066; hydroxyfomononetin, 283.061; C15H10O5, 269.046; C17H14O5, 297.077; glyceollin, 337.108. Statistically significant differences were determined by two-tailed Student’s *t*-test (^∗^*p* < 0.05; ^∗∗^*p* < 0.005; *n* = 7–9).

Defense mechanisms against aphids can be phloem-based or can be effected by metabolites that modify aphid feeding behavior before the insects reach the sieve elements (Walling and Thompson, 2012). To determine where isoflavonoids carry out their defensive role against soybean aphid, we took advantage of a mass spectrometry (MS) imaging technique recently optimized for this system (Klein et al., 2015). This technique allowed us to create 2D maps of metabolite distributions on leaf, by directly analyzing leaf compounds from imprints made by pressing soybean leaves on a porous Teflon surface. We observed a significant accumulation of different isoflavones, including formononetin, daidzein, and glyceollin in leaves colonized by aphid, while almost no signal for these compounds was detected in control plants (Figure 7). Quantification of the relative MS signal for each compound confirmed that isoflavones significantly accumulate in aphid-fed leaves (Figure 8). In addition, we were able to determine that this accumulation does not occur in the leaf vasculature. The vasculature is clearly discernible in the optical image for the imprinting of an aphid-infested leaf in Figure 7 (lower left panel), and it is also clear in the panels corresponding to individual isoflavones that the MS signals are not located in the vascular tissue. Rather, they likely accumulate in parenchyma or epidermal cells, with higher accumulation closer to the vascular tissue. This distribution is unlikely to be the result of an artifact due to imprinting. As we have previously demonstrated, the imprinting process does not induce broadening any more than 20–30 μm or less, and metabolites localized in the vasculature can be easily visualized (see for example salicylic acid localization in Figure 4 of Klein et al., 2015).

## Discussion

To our knowledge, this is the first transcriptome analysis of the soybean response to long-term (21 days) aphid colonization, and it complements previous studies that analyzed susceptible and *Rag1-*dependent resistant soybean responses to early [6 and 12 h (Li et al., 2008)] and mid-range [1 and 7 days (Studham and MacIntosh, 2013)] aphid feeding, and also transcriptome analyses of *Rag2-* and *Rag5-* carrying lines at early and mid-range time points [up to 48 h of aphid feeding (Brechenmacher et al., 2015; Lee et al., 2017)].

As in our previous transcriptome analysis, there were no significant transcriptional differences between aphid-infested and uninfested resistant plants with the *Rag1* gene after 21-day feeding, yet aphid population growth was clearly slower in these plants compared to the susceptible variety. However, the number of aphids on resistant plants at day 21 is comparable to the number of aphid observed on susceptible plants after 7 days of infestation [compare with results in Studham and MacIntosh (2013)]; and susceptible plants mount a strong transcriptional response to aphid feeding at 7 days. Thus, resistant plants can withstand an aphid load that triggers significant responses in S plants without the need to reprogram their transcriptome. The lack of R response suggests the presence of constitutive resistance, as proposed previously for *Rag1* and *Rag5* resistance (Studham and MacIntosh, 2013; Lee et al., 2017), and evidenced by DE transcripts between uninfested S and R plants. Evidence of constitutive differences between the same susceptible and *Rag1* plants used here was also obtained through amino acid profiling of plants grown in field conditions and subject to natural aphid infestations (Chiozza et al., 2010). Constitutive resistance has also been proposed for other aphid resistant lines in other species, including aphid resistance in wheat (Han et al., 2009) and barley (Delp et al., 2009).

The higher levels of several ferritin transcripts in R plants could be part of the constitutive resistance mechanism. Similar results were reported by Li et al. (2008), who found constitutively higher levels of *ferritin-1* and *ferritin-2* transcripts in Dowling (*Rag1*) compared with Williams 82 (S) in the absence of aphids, and a further increase in *ferritin* expression levels in aphid-infested plants. A transcriptome analysis of soybean plants tolerant to aphid infestation identified differential expression of an iron transporter and ferritins, also suggesting that iron homeostasis may be relevant in soybean-soybean aphid interactions (Prochaska et al., 2015). Once an infection or infestation is established, pest and plant compete for the plant endogenous resources, such as iron. Ferritins are protein used by plants and pathogens to store iron, and thus an increase in the expression of genes encoding ferritins could suggest the presence in R plants of a mechanism to sequester iron and limit its availability for the insect, as has been suggested for some plant–microbe interactions (Briat et al., 2010). For example, plant ferritin transcripts are induced by pathogen attack in Arabidopsis, and the lack of a functional ferritin gene (*AtFer1*) results in enhanced susceptibility to a pathogenic bacterium (Dellagi et al., 2005). On the other hand, the bacterial soft rot (*Erwinia chrysanthemi*) expresses ferritins that can successfully compete with soybean ferritin to obtain iron from a soybean cell suspension (Neema et al., 1993).

There is, however, a difference in the number of DE transcripts in the uninfested R vs. S plant comparison between the 1 and 7 day microarray experiment (Studham and MacIntosh, 2013) and the current analysis. The lower number of DE transcripts in the 21 day transcriptome could be explained by differential airborne priming. The aphid-infested and uninfested plants in both experiments were grown in the same growth chamber, thus it is likely that airborne priming signals from the infested plants were detected by the uninfested plants. This primed state involves transcriptional changes that enable the uninfested plants to be more sensitive to defense related signals in the event of an attack. However, there is a significant fitness cost to maintaining a primed state, and the induction of defense responses during priming is transient (Martinez-Medina et al., 2016). Although there are no reports on the duration of soybean priming, it seems unlikely that the plant would remain in a primed state 21 days after the onset of the infestation in neighboring plants. In fact, a reduction in the number of DE genes in the uninfested comparison was observed from the 1 to 7 day, and from 7 to 21 day responses. This observation also suggests that R plants are able to induce a stronger priming response (Studham and MacIntosh, 2013).

We observed a significant induction of defense programs in S plants, and the defense transcriptional response seems to be dominated by a PTI program. In particular, key regulators of PTI are induced in response to long-term aphid feeding, including orthologs of *MPK3* and *WRKY33* that are upregulated almost 5- and 6- fold respectively. The MAPK phosphorylation cascade is recognized as a central regulator of plant innate immunity (Asai et al., 2002; Bigeard et al., 2015). Activated MPK3 phosphorylates WRKY33 that in turn regulates phytoalexins production in Arabidopsis (Mao et al., 2011). Similarly, a PAMP-induced phosphorylation cascade that leads to the activation of the MPK3/6 in soybean has been proposed as a regulator for production of glyceollins (Daxberger et al., 2007). More significant, *TaWRKY53*, the wheat ortholog of *AtWRKY33* is also induced by aphid infestation in an aphid-resistant wheat line (Botha et al., 2010), and silencing of this gene results in a reduction in expression of a phenylalanine ammonia-lyase (*PAL*) gene an increase in susceptibility to aphid infestation (Van Eck et al., 2010). Interestingly, the same MPK3/WRKY33 module has been implicated in the induction of PTI in response to chitin in Arabidopsis (Son et al., 2011) as part of a defense network that includes another transcription factor, WRKY40, also found in our list of DE genes. Almost 27% of the DE genes identified in the S response in our analysis are annotated as responsive to chitin, and “response to chitin” is the most overrepresented GO category in this dataset. Given the similarities between the S response and chitin-triggered PTI, it is probable that chitin acts as an important HAMPs in the aphid–soybean interaction. Given the already well-established role of chitin as a PAMP and the presence of chitin in the aphid exoskeleton and stylet, the possibility that chitin acts as HAMP has already been proposed (Whiteman et al., 2011; Van Eck et al., 2014; Kaloshian and Walling, 2016; Lee et al., 2017); our results provide experimental support to this hypothesis.

Plant innate immunity triggered by PAMP results in the activation of an immune secretory pathway that facilitates deployment of defense mechanisms such as callose deposition, cell wall modifications, and release of phytoalexins (Collins et al., 2003; Humphry et al., 2010; Yun et al., 2016). Our S dataset contained a number of induced DE genes orthologs to the syntaxin PEN1 and other SNARE domain proteins, as well as DE genes corresponding to several cell wall modifying enzymes including expansins, xyloglucan endotransglycosylases, cellulose synthases, and others. In soybean, this immune secretory pathway is an important component of the defense response against soybean cyst nematodes (Klink et al., 2017). An ortholog of the Arabidopsis TF MYB15, which controls lignin production in response to pathogens (Chezem et al., 2017), is induced more than fivefold in the S response. Our results strongly suggest that long-term exposure to aphid feeding triggers a HAMP-regulated response that result in cell wall modifications likely to reduce aphid stylet penetration.

In addition to these modifications, the S response showed a significant induction of the phenylpropanoid pathway. While this pathway could be activated as part of the cell wall strengthening response, our results showed that one important outcome of this activation is the accumulation of isoflavone phytoalexins. We also showed that isoflavones accumulate in leaves colonized by aphids, likely in the parenchyma, and at least daidzein has a deterrent effect against the insect. A role for isoflavones was also hypothesized in incompatible soybean-soybean aphid interactions. Li et al. (2008) observed induction of *Glyma.08g109500*, a chalcone synthase, in the early *Rag1* response to aphids, and the same gene was DE in our susceptible 21-day response. Moreover, an *A. glycines* transcriptome analysis found that a large proportion of the genes DE in soybean aphids feeding on *Rag1* resistant plants encoded proteins associated with detoxifying mechanisms, a pattern typical of xenobiotic stress response (Bansal et al., 2014). This result suggests that the antibiosis effect observed for *Rag1* may be mediated by toxic secondary metabolites, and is consistent with a defensive role for isoflavones as proposed by Li et al. (2008) and by our experiments. The identification of soybean aphid resistance QTLs that map to loci in the soybean genome that are also highly associated with high isoflavone content provides another support for isoflavone-mediated aphid defense mechanisms (Meng et al., 2011).

Isoflavones are well-known for their antimicrobial activities, and in soybean the main isoflavone phytoalexins are glyceollins (Hammerschmidt, 1999; Dixon and Pasinetti, 2010). However, the role of these compounds in defense against herbivores has not been extensively characterized. More importantly, other isoflavones, normally considered just precursors of glyceollins, could be active deterrents or insecticidal compounds. Using artificial diets, Goławska and Łukasik (2012) showed that genistein has antifeedant activity against the pea aphid (*Acyrthosiphon pisum*). Resistance to other sucking insects may also correlate with isoflavone content. For example, induction of isoflavone accumulation by exposure to UV light resulted in an increase in resistance to the stink bugs *Nezara viridula* and *Piezodorus guildinii* in soybean (Zavala et al., 2015). *N. viridula* feeding also induced accumulation of daidzin and genistin (glucoside derivatives of daidzein and genistein) in soybean pods, and extracts of injured pods had a deterrent effect on feeding (Piubelli et al., 2003).

A role for isoflavones in defense against other insect guilds, particularly Lepidoptera, has also been proposed. For example, the isoflavones daidzin, 4′,7-dihydroxyflavone, daidzein, and formononetin accumulated in soybean leaves in response to feeding by *Spodoptera litura* or after application of *S. litura* oral secretions (Murakami et al., 2014). Interestingly, glyceollins were not detected in this interaction, and the authors suggested that the accumulating isoflavones are active compounds against insects, and not just intermediate compounds in glyceollin biosynthesis (Murakami et al., 2014), although it is not clear whether the lack of glyceollin detection is due to a true absence of the compounds or a limitation of the technique used. A different analysis of phytoalexins produced in response to *S. litura* also identified daidzein as the main isoflavone accumulating in soybean leaves, although a small amount of different glyceollins was also identified in this analysis (Zhou et al., 2011). A characterization of different soybean varieties identified a correlation between genistein and rutin (a flavonoid) concentrations and reduced *Anticarsia gemmatalis* larval growth (Piubelli et al., 2005). Feeding experiments with isoflavones extracted from the insect-resistant soybean cultivar PI227687 determined that daidzein had antifeedant and/or antibiotic effects against *Trichoplusia ni*, and it was more effective than glyceollins (Sharma and Norris, 1991). In a different feeding experiment Zhou et al. (2011) determined that daidzein could significantly reduce *S. litura* larval growth, while genistein and genistin had no effect. Analysis of insect frass indicated that daidzein was not metabolized by the insect, while the other two isoflavones were processed in the insect gut (Zhou et al., 2011). A classical mosquito larval toxicity bioassay also identified genistein, daidzein, and acetate derivatives of these compounds as insecticidal (Rao et al., 1990). These findings correlate well with our results and could suggest that isoflavones other than glyceollins may be the active components of soybean chemical defenses against insects. However, it is also important to consider that different compounds could be toxic at very different concentrations, and thus a role for glyceollins cannot be excluded.

Through their path to reach the phloem, aphids encounter several cell layers, and plants have evolved different mechanisms of surface resistance, epidermis resistance, mesophyll resistance, and phloem resistance to stop or slow down aphid feeding (Alvarez et al., 2006; Walling and Thompson, 2012). Studies of stylet sheath path and electropenetration graph analyses of aphid feeding have determined that aphids puncture and “taste” all mesophyll cells that are encountered during the probing phase until they reach a sieve element (Tjallingii, 2006; Walling, 2008). The accumulation of allelochemicals has been previously associated with mesophyll defenses and antifeedant activities (Corcuera et al., 1992; Gabrys and Tjallingii, 2002; Kordan et al., 2012). Our results also suggest that soybean accumulates isoflavones phytoalexins in mesophyll cells as a mechanism of defense against aphids, deterring feeding before aphids reach the phloem.

Our study indicates that susceptible soybean plants have the ability to deploy effective defenses against aphids. Thus, why are these defenses not expressed earlier, when aphids are successfully colonizing the plant? Based on our previous transcriptome and fatty acid analyses of the plant response after 1 or 7 days of aphid feeding, we proposed that aphids induce an ABA-dependent decoy response that antagonizes effective SA- and JA-dependent defenses (Studham and MacIntosh, 2013; Kanobe et al., 2015). Interestingly, JA treatments enhance isoflavones accumulation in soybean in response to elicitors, while ABA treatments inhibit glucan elicitor-induced isoflavone accumulation (Graham and Graham, 1996). Thus, the strong induction of ABA biosynthesis and signaling observed at 7 days of aphid feeding would block production of isoflavones and reduce the ability of susceptible soybean plants to inhibit aphid feeding. A similar ABA-mediated block in the accumulation of allelochemical defenses has been proposed in compatible *Myzus persicae* – Arabidopsis interactions (Hillwig et al., 2016); *M. persicae* performs better on ABA biosynthesis mutants than on WT plants and the ABA deficient plants accumulate more indole glucosinolates known to have antibiotic effects on aphids. On the other hand, the soybean response to aphids after 21 days of feeding does not display an ABA response, suggesting that the plant is able to eventually escape from the aphid-induced defense suppression, but only after aphids have heavily colonized the plant.

An alternative hypothesis considered to explain the increase in ABA signaling after 7 days of aphid feeding in soybean and Arabidopsis could be that aphids cause water stress in soybean, as has been proposed in other plant aphid-interaction (Cabrera et al., 1995). However, the aphid population established after 21 days was 20-fold higher than at 7 days, and it would be expected that any water stress caused by aphids would be directly proportional to their number; yet no ABA response was observed at 21 days, arguing against a water stress response. In fact, the ∼5-fold increase in the expression of the orthologs of *AtWRKY33* and *AtHOS3* (*Glyma.01G128100* and *Glyma.04G034400*, respectively; Table 1 and Supplementary Table 1) in the 21 day response would suggest an active repression of ABA signaling. Arabidopsis *hos3* mutants are hypersensitive to osmotic stress and other abiotic stresses, and it has been proposed that HOS3 is a negative regulator of ABA-dependent processes (Quist et al., 2009). AtWRKY33 seems to act as a transcriptional inhibitor of *NCED3* and *NCED5*, two ABA biosynthesis genes, and the negative regulation of ABA signaling by AtWRKY33 is necessary for immunity against the necrotrophic fungus *Botrytis cinerea* (Liu et al., 2015). Interestingly, the promoter regions of the wheat and rice orthologs of *AtWRKY3*3 (*TaWRKY53* and *OsWRKY53*) have conserved ABRE motifs, suggesting a potential regulation by ABA (Van Eck et al., 2014), and could indicate a point of crosstalk between PTI and ABA signaling. Our data indirectly support the hypothesis that, during compatible interactions, aphids use the plant’s ABA pathway to repress effective defenses during the colonization process, but the plant can eventually overcome this suppression and activate a PTI response leading to the production of phytoalexins to reduce aphid feeding.

Currently, it is not clear whether these late defense mechanisms are the result of a plant desensitization to aphid effectors that suppress defenses, or the accumulation of high level of HAMP signals due to the elevated number of aphids colonizing the plant. It is even possible to imagine that aphids suppress the production of their own effectors that interfere with plant defenses in response to crowding; thus inducing their migration to other, uninfested plants as a consequence of the now unrestricted increase in feeding deterrents. More research is needed to test these hypotheses and understand this dynamic plant–insect interaction.

## Data Availability

The datasets generated for this study can be found in NCBI’s Gene Expression Omnibus, GSE115790.

## Author Contributions

JDH, MES, and GCM designed the study. JDH, MES, AK, NK, KB, Y-JL, and GCM performed experiments and/or analyzed the data. MES and GCM wrote the manuscript. JDH, NK, and Y-JL revised the text. All authors read and approved the final manuscript.

## Conflict of Interest Statement

The authors declare that the research was conducted in the absence of any commercial or financial relationships that could be construed as a potential conflict of interest.

## Footnotes

1 https://www.ncbi.nlm.nih.gov/geo/query/acc.cgi?acc=GSE115790

## Supplementary Material

TABLE S1 Differentially expressed genes for susceptible and resistant responses to 21 days of aphid feeding, and genetic differences between susceptible and resistant plants.

TABLE S2 GO-term analysis for each DE gene dataset.

TABLE S3 Differentially expressed gene set analysis (GSA).

TABLE S4 Ferritin genes differentially expressed in resistant plants.

## References

[B1] Akashi T. Aoki T. Ayabe S. (2005). Molecular and biochemical characterization of 2-hydroxyisoflavanone dehydratase. involvement of carboxylesterase-like proteins in leguminous isoflavone biosynthesis. *Plant Physiol.* 137 882–891. 10.1104/pp.104.056747 15734910 PMC1065389

[B2] Akashi T. Sasaki K. Aoki T. Ayabe S. Yazaki K. (2009). Molecular cloning and characterization of a cDNA for pterocarpan 4-dimethylallyltransferase catalyzing the key prenylation step in the biosynthesis of glyceollin, a soybean phytoalexin. *Plant Physiol.* 149 683–693. 10.1104/pp.108.123679 19091879 PMC2633842

[B3] Altschul S. F. Gish W. Miller W. Myers E. W. Lipman D. J. (1990). Basic local alignment search tool. *J. Mol. Biol.* 215 403–410. 10.1016/S0022-2836(05)80360-2 2231712

[B4] Alvarez A. E. Tjallingii W. F. Garzo E. Vleeshouwers V. Dicke M. Vosman B. (2006). Location of resistance factors in the leaves of potato and wild tuber-bearing *Solanum* species to the aphid *Myzus persicae*. *Entomol. Exp. Et Appl.* 121 145–157. 10.1111/j.1570-8703.2006.00464.x

[B5] Asai T. Tena G. Plotnikova J. Willmann M. R. Chiu W.-L. Gomez-Gomez L. (2002). MAP kinase signalling cascade in *Arabidopsis* innate immunity. *Nature* 415 977–983. 10.1038/415977a 11875555

[B6] Asselbergh B. De Vleesschauwer D. Hofte M. (2008). Global switches and fine-tuning - ABA modulates plant pathogen defense. *Mol. Plant Microbe Interact.* 21 709–719. 10.1094/mpmi-21-6-0709 18624635

[B7] Bakshi M. Oelmüller R. (2014). WRKY transcription factors. *Plant Signal. Behav.* 9:e27700 10.4161/psb.27700 PMC4091213 24492469

[B8] Bansal R. Mian M. Mittapalli O. Michel A. P. (2014). RNA-Seq reveals a xenobiotic stress response in the soybean aphid, *Aphis glycines*, when fed aphid-resistant soybean. *BMC Genomics* 15:972. 10.1186/1471-2164-15-972 25399334 PMC4289043

[B9] Bari R. Jones J. (2009). Role of plant hormones in plant defence responses. *Plant Mol. Biol.* 69 473–488. 10.1007/s11103-008-9435-0 19083153

[B10] Bigeard J. Colcombet J. Hirt H. (2015). Signaling mechanisms in pattern-triggered immunity (PTI). *Mol. Plant* 8 521–539. 10.1016/j.molp.2014.12.022 25744358

[B11] Bomati E. K. Austin M. B. Bowman M. E. Dixon R. A. Noel J. P. (2005). Structural elucidation of chalcone reductase and implications for deoxychalcone biosynthesis. *J. Biol. Chem.* 280 30496–30503. 10.1074/jbc.M502239200 15970585 PMC2860619

[B12] Botha A.-M. Swanevelder Z. H. Lapitan N. L. V. (2010). Transcript profiling of wheat genes expressed during feeding by two different biotypes of *Diuraphis noxia*. *Environ. Entomol.* 39 1206–1231. 10.1603/en09248 22127172

[B13] Boudsocq M. Willmann M. R. McCormack M. Lee H. Shan L. He P. (2010). Differential innate immune signalling via Ca2+ sensor protein kinases. *Nature* 464:418. 10.1038/nature08794 20164835 PMC2841715

[B14] Brechenmacher L. Nguyen T. H. N. Zhang N. Jun T.-H. Xu D. Mian M. A. R. (2015). Identification of soybean proteins and genes differentially regulated in near isogenic lines differing in resistance to aphid infestation. *J. Proteome Res.* 14 4137–4146. 10.1021/acs.jproteome.5b00146 26350764

[B15] Briat J.-F. Ravet K. Arnaud N. Duc C. Boucherez J. Touraine B. (2010). New insights into ferritin synthesis and function highlight a link between iron homeostasis and oxidative stress in plants. *Ann. Bot.* 105 811–822. 10.1093/aob/mcp128 19482877 PMC2859905

[B16] Cabrera H. M. Argandoña V. H. Zúñiga G. E. Corcuera L. J. (1995). Effect of infestation by aphids on the water status of barley and insect development. *Phytochemistry* 40 1083–1088. 10.1016/0031-9422(95)00325-2

[B17] Caspi R. Altman T. Dale J. M. Dreher K. Fulcher C. A. Gilham F. (2009). The MetaCyc database of metabolic pathways and enzymes and the bioCyc collection of pathway/genome databases. *Nucleic Acids Res.* 38 D473–D479. 10.1093/nar/gkp875 19850718 PMC2808959

[B18] Chezem W. R. Memon A. Li F.-S. Weng J.-K. Clay N. K. (2017). SG2-Type R2R3-MYB transcription factor MYB15 controls defense-induced lignification and basal immunity in *Arabidopsis*. *Plant Cell* 29 1907–1926. 10.1105/tpc.16.00954 28733420 PMC5590497

[B19] Chiozza M. V. O’Neal M. E. MacIntosh G. C. (2010). Constitutive and induced differential accumulation of amino acid in leaves of susceptible and resistant soybean plants in response to the soybean aphid (*Hemiptera: Aphididae*). *Environ. Entomol.* 39 856–864. 10.1603/en09338 20550799

[B20] Collins N. C. Thordal-Christensen H. Lipka V. Bau S. Kombrink E. Qiu J.-L. (2003). SNARE-protein-mediated disease resistance at the plant cell wall. *Nature* 425:973. 10.1038/nature02076 14586469

[B21] Corcuera L. J. Argandoña V. H. Zúñiga G. E. (1992). “Allelochemicals in wheat and barley: role in plant—insect interactions,” in *Allelopathy: Basic and Applied Aspects*, eds Rizvi S. J. H. Rizvi V. (Dordrecht: Springer), 119–127.

[B22] Daxberger A. Nemak A. Mithöfer A. Fliegmann J. Ligterink W. Hirt H. (2007). Activation of members of a MAPK module in β-glucan elicitor-mediated non-host resistance of soybean. *Planta* 225 1559–1571. 10.1007/s00425-006-0442-6 17123101

[B23] De Vos M. Van Oosten V. R. Van Poecke R. M. P. Van Pelt J. A. Pozo M. J. Mueller M. J. (2005). Signal signature and transcriptome changes of *Arabidopsis* during pathogen and insect attack. *Mol. Plant Microbe Interact.* 18 923–937. 10.1094/mpmi-18-0923 16167763

[B24] Deavours B. E. Liu C. J. Naoumkina M. A. Tang Y. Farag M. A. Sumner L. W. (2006). Functional analysis of members of the isoflavone and isoflavanone O-methyltransferase enzyme families from the model legume *Medicago truncatula*. *Plant Mol. Biol.* 62 715–733. 10.1007/s11103-006-9050-x 17001495 PMC2862459

[B25] Dellagi A. Rigault M. Segond D. Roux C. Kraepiel Y. Cellier F. (2005). Siderophore-mediated upregulation of *Arabidopsis ferritin* expression in response to *Erwinia chrysanthemi* infection. *Plant J.* 43 262–272. 10.1111/j.1365-313X.2005.02451.x 15998312

[B26] Delp G. Gradin T. Åhman I. Jonsson L. (2009). Microarray analysis of the interaction between the aphid *Rhopalosiphum padi* and host plants reveals both differences and similarities between susceptible and partially resistant barley lines. *Mol. Genet. Genomics* 281 233–248. 10.1007/s00438-008-0409-3 19085010

[B27] Dixon R. A. Pasinetti G. M. (2010). Flavonoids and isoflavonoids: from plant biology to agriculture and neuroscience. *Plant Physiol.* 154 453–457. 10.1104/pp.110.161430 20921162 PMC2948995

[B28] Edgar R. Domrachev M. Lash A. E. (2002). Gene expression omnibus: NCBI gene expression and hybridization array data repository. *Nucleic Acids Res.* 30 207–210. 10.1093/nar/30.1.207 11752295 PMC99122

[B29] Efron B. Tibshirani R. (2007). On testing the significance of sets of genes. *Ann. Appl. Stat.* 1 107–129. 10.1214/07-AOAS101

[B30] Farrell K. Jahan M. Kovinich N. (2017). Distinct Mechanisms of biotic and chemical elicitors enable additive elicitation of the anticancer phytoalexin glyceollin I. *Molecules* 22:1261. 10.3390/molecules22081261 28749423 PMC6152012

[B31] Fliegmann J. Furtwangler K. Malterer G. Cantarello C. Schuler G. Ebel J. (2010). Flavone synthase II (CYP93B16) from soybean (*Glycine max* L.). *Phytochemistry* 71 508–514. 10.1016/j.phytochem.2010.01.007 20132953

[B32] Gabrys B. Tjallingii W. F. (2002). The role of sinigrin in host plant recognition by aphids during initial plant penetration. *Entomol. Exp. Et Appl.* 104 89–93. 10.1046/j.1570-7458.2002.00994.x

[B33] Giordanengo P. Brunissen L. Rusterucci C. Vincent C. van Bel A. Dinant S. (2010). Compatible plant-aphid interactions: how aphids manipulate plant responses. *Comp. Rend. Biol.* 333 516–523. 10.1016/j.crvi.2010.03.007 20541163

[B34] Goggin F. L. (2007). Plant-aphid interactions: molecular and ecological perspectives. *Curr. Opin. Plant Biol.* 10 399–408. 10.1016/j.pbi.2007.06.004 17652010

[B35] Goławska S. Łukasik I. (2012). Antifeedant activity of luteolin and genistein against the pea aphid. *Acyrthosiphon pisum*. *J. Pest. Sci.* 85 443–450. 10.1007/s10340-012-0452-z 23204991 PMC3505511

[B36] Graham T. L. Graham M. Y. (1996). Signaling in soybean phenylpropanoid responses (dissection of primary, secondary, and conditioning effects of light, wounding, and elicitor treatments). *Plant Physiol.* 110 1123–1133. 10.1104/pp.110.4.1123 12226246 PMC160894

[B37] Grant D. Nelson R. T. Cannon S. B. Shoemaker R. C. (2010). SoyBase, the USDA-ARS soybean genetics and genomics database. *Nucleic Acids Res.* 38(Suppl. 1), D843–D846. 10.1093/nar/gkp798 20008513 PMC2808871

[B38] Hammerschmidt R. (1999). PHYTOALEXINS: what have we learned after 60 years? *Ann. Rev. Phytopathol.* 37 285–306. 10.1146/annurev.phyto.37.1.285 11701825

[B39] Han Y. Wang Y. Bi J.-L. Yang X.-Q. Huang Y. Zhao X. (2009). Constitutive and induced activities of defense-related enzymes in aphid-resistant and aphid-susceptible cultivars of wheat. *J. Chem. Ecol.* 35 176–182. 10.1007/s10886-009-9589-5 19159980

[B40] Hesler L. S. Chiozza M. V. O’Neal M. E. MacIntosh G. C. Tilmon K. J. Chandrasena D. I. (2013). Performance and prospects of Rag genes for management of soybean aphid. *Entomol. Exp. Et Appl.* 147 201–216. 10.1111/eea.12073

[B41] Hesler L. S. Dashiell K. E. (2007). Resistance to *Aphis glycines* (*Hemiptera: aphididae*) in various soybean lines under controlled laboratory conditions. *J. Econ. Entomol.* 100 1464–1469. 10.1093/jee/100.4.1464 17849903

[B42] Hill C. B. Li Y. Hartman G. L. (2006a). A single dominant gene for resistance to the soybean aphid in the soybean cultivar dowling. *Crop. Sci.* 46 1601–1605. 10.2135/cropsci2005.11-0421

[B43] Hill C. B. Li Y. Hartman G. L. (2006b). Soybean aphid resistance in soybean jackson is controlled by a single dominant gene. *Crop. Sci.* 46 1606–1608. 10.2135/cropsci2005.11-0438

[B44] Hill J. H. Alleman R. Hogg D. B. Grau C. R. (2001). First Report of transmission of soybean mosaic virus and alfalfa mosaic virus by *Aphis glycines* in the new world. *Plant Dis.* 85:561.3. 10.1094/pdis.2001.85.5.561c 30823148

[B45] Hillwig M. S. Chiozza M. Casteel C. L. Lau S. T. Hohenstein J. Hernández E. (2016). Abscisic acid deficiency increases defence responses against *Myzus persicae* in *Arabidopsis*. *Mol. Plant Pathol.* 17 225–235. 10.1111/mpp.12274 25943308 PMC6638517

[B46] Hogenhout S. A. Bos J. I. B. (2011). Effector proteins that modulate plant–insect interactions. *Curr. Opin. Plant Biol.* 14 422–428. 10.1016/j.pbi.2011.05.003 21684190

[B47] Howe G. A. Jander G. (2008). Plant immunity to insect herbivores. *Ann. Rev. Plant Biol.* 59 41–66. 10.1146/annurev.arplant.59.032607.092825 18031220

[B48] Humphry M. Bednarek P. Kemmerling B. Koh S. Stein M. Göbel U. (2010). A regulon conserved in monocot and dicot plants defines a functional module in antifungal plant immunity. *Proc. Natl. Acad. Sci.* 107 21896–21901. 10.1073/pnas.1003619107 21098265 PMC3003077

[B49] Irizarry R. A. Hobbs B. Collin F. Beazer-Barclay Y. D. Antonellis K. J. Scherf U. (2003). Exploration, normalization, and summaries of high density oligonucleotide array probe level data. *Biostatistics* 4 249–264. 10.1093/biostatistics/4.2.249 12925520

[B50] Jaouannet M. Rodriguez P. A. Thorpe P. Lenoir C. J. G. MacLeod R. Escudero-Martinez C. (2014). Plant immunity in plant-aphid interactions. *Front. Plant Sci.* 5:10. 10.3389/fpls.2014.00663 25520727 PMC4249712

[B51] Jung W. Yu O. Lau S. M. O’Keefe D. P. Odell J. Fader G. (2000). Identification and expression of isoflavone synthase, the key enzyme for biosynthesis of isoflavones in legumes. *Nat. Biotechnol.* 18 208–212. 10.1038/72671 10657130

[B52] Kaloshian I. Walling L. L. (2016). “Plant Immunity: Connecting the Dots Between Microbial and Hemipteran Immune Responses,” in *Management of Insect Pests to Agriculture: Lessons Learned from Deciphering their Genome, Transcriptome and Proteome*, eds Czosnek H. Ghanim M. (Cham: Springer International Publishing), 217–243.

[B53] Kamphuis L. G. Zulak K. Gao L. L. Anderson J. Singh K. B. (2013). Plant-aphid interactions with a focus on legumes. *Funct. Plant Biol.* 40 1271–1284. 10.1071/fp1309 32481194

[B54] Kanobe C. McCarville M. T. O’Neal M. E. Tylka G. L. MacIntosh G. C. (2015). Soybean aphid infestation induces changes in fatty acid metabolism in soybean. *PLoS One* 10:e0145660. 10.1371/journal.pone.0145660 26684003 PMC4684210

[B55] Kim D. H. Kim B.-G. Lee Y. Ryu J. Y. Lim Y. Hur H.-G. (2005). Regiospecific methylation of naringenin to ponciretin by soybean O-methyltransferase expressed in *Escherichia coli*. *J. Biotechnol.* 119 155–162. 10.1016/j.jbiotec.2005.04.004 15961179

[B56] Kim K.-S. Bellendir S. Hudson K. Hill C. Hartman G. Hyten D. (2010). Fine mapping the soybean aphid resistance gene Rag1 in soybean. *Theor. Appl. Genet.* 120 1063–1071. 10.1007/s00122-009-1234-8 20035316

[B57] Kim K.-S. Hill C. B. Hartman G. L. Mian M. A. R. Diers B. W. (2008). Discovery of soybean aphid biotypes. *Crop. Sci.* 48 923–928. 10.2135/cropsci2007.08.0447

[B58] Klein A. T. Yagnik G. B. Hohenstein J. D. Ji Z. Zi J. Reichert M. D. (2015). Investigation of the chemical interface in the soybean–aphid and rice–bacteria interactions using maldi-mass spectrometry imaging. *Anal. Chem.* 87 5294–5301. 10.1021/acs.analchem.5b00459 25914940

[B59] Klink V. P. Sharma K. Pant S. R. McNeece B. Niraula P. Lawrence G. W. (2017). Components of the SNARE-containing regulon are co-regulated in root cells undergoing defense. *Plant Signal. Behav.* 12:e1274481. 10.1080/15592324.2016.1274481 28010187 PMC5351740

[B60] Kordan B. Dancewicz K. Wróblewska A. Gabryś B. (2012). Intraspecific variation in alkaloid profile of four lupine species with implications for the pea aphid probing behaviour. *Phytochem. Lett.* 5 71–77. 10.1016/j.phytol.2011.10.003

[B61] Lee S. Cassone B. J. Wijeratne A. Jun T.-H. Michel A. P. Mian M. A. R. (2017). Transcriptomic dynamics in soybean near-isogenic lines differing in alleles for an aphid resistance gene, following infestation by soybean aphid biotype 2. *BMC Genomics* 18:472. 10.1186/s12864-017-3829-9 28645245 PMC5481885

[B62] Li Y. Hill C. B. Carlson S. R. Diers B. W. Hartman G. L. (2007). Soybean aphid resistance genes in the soybean cultivars dowling and Jackson map to linkage group M. *Mol. Breed.* 19 25–34. 10.1007/s11032-006-9039-9

[B63] Li Y. Zou J. Li M. Bilgin D. D. Vodkin L. O. Hartman G. L. (2008). Soybean defense responses to the soybean aphid. *N. Phytol.* 179 185–195. 10.1111/j.1469-8137.2008.02443.x 18422900

[B64] Liu S. Kracher B. Ziegler J. Birkenbihl R. P. Somssich I. E. (2015). Negative regulation of ABA signaling by WRKY33 is critical for *Arabidopsis* immunity towards *Botrytis cinerea* 2100. *eLife* 4:e07295. 10.7554/eLife.07295 26076231 PMC4487144

[B65] Mao G. Meng X. Liu Y. Zheng Z. Chen Z. Zhang S. (2011). Phosphorylation of a WRKY transcription factor by two pathogen-responsive MAPKs drives phytoalexin biosynthesis in em *Arabidopsis* em. *Plant Cell* 23 1639–1653. 10.1105/tpc.111.084996 21498677 PMC3101563

[B66] Martinez-Medina A. Flors V. Heil M. Mauch-Mani B. Pieterse C. M. J. Pozo M. J. (2016). Recognizing plant defense priming. *Trends Plant Sci.* 21 818–822. 10.1016/j.tplants.2016.07.009 27507609

[B67] McGinnis S. Madden T. L. (2004). BLAST: at the core of a powerful and diverse set of sequence analysis tools. *Nucleic Acids Res.* 32 W20–W25. 10.1093/nar/gkh435 15215342 PMC441573

[B68] Meng F. Han Y. Teng W. Li Y. Li W. (2011). QTL underlying the resistance to soybean aphid (*Aphis glycines* matsumura) through isoflavone-mediated antibiosis in soybean cultivar ‘zhongdou 27’. *Theor. Appl. Genet.* 123 1459–1465. 10.1007/s00122-011-1680-y 21858470

[B69] Mensah C. DiFonzo C. Wang D. (2008). Inheritance of soybean aphid resistance in PI 567541B and PI 567598B. *Crop. Sci.* 48 1759–1763. 10.2135/cropsci2007.09.0535

[B70] Mithöfer A. Boland W. (2008). Recognition of herbivory-associated molecular patterns. *Plant Physiol.* 146 825–831. 10.1104/pp.107.113118 18316636 PMC2259064

[B71] Morales A. M. A. P. O’Rourke J. A. van de Mortel M. Scheider K. T. Bancroft T. J. Borém A. (2013). Transcriptome analyses and virus induced gene silencing identify genes in the Rpp4-mediated Asian soybean rust resistance pathway. *Funct. Plant Biol.* 40 1029–1047. 10.1071/FP12296 32481171

[B72] Morkunas I. Mai V. Gabrys B. (2011). Phytohormonal signaling in plant responses to aphid feeding. *Acta Physiol. Plant.* 33 2057–2073. 10.1007/s11738-011-0751-7

[B73] Murakami S. Nakata R. Aboshi T. Yoshinaga N. Teraishi M. Okumoto Y. (2014). Insect-induced daidzein, formononetin and their conjugates in soybean leaves. *Metabolites* 4:532. 10.3390/metabo4030532 25000357 PMC4192678

[B74] Nagamatsu A. Masuta C. Senda M. Matsuura H. Kasai A. Hong J. S. (2007). Functional analysis of soybean genes involved in flavonoid biosynthesis by virus-induced gene silencing. *Plant Biotechnol. J.* 5 778–790. 10.1111/j.1467-7652.2007.00288.x 17764520

[B75] Neema C. Laulhere J. P. Expert D. (1993). Iron deficiency induced by chrysobactin in *Saintpaulia* leaves Inoculated with *Erwinia chrysanthemi*. *Plant Physiol.* 102 967–973. 10.1104/pp.102.3.967 12231882 PMC158870

[B76] Nuruzzaman M. Sharoni A. M. Kikuchi S. (2013). Roles of NAC transcription factors in the regulation of biotic and abiotic stress responses in plants. *Front. Microbiol.* 4:248 10.3389/fmicb.2013.00248 PMC3759801 24058359

[B77] Pfaffl M. W. (2001). A new mathematical model for relative quantification in real-time RT-PCR. *Nucleic Acids Res.* 29:e45 10.1093/nar/29.9.e45 PMC55695 11328886

[B78] Pieterse C. M. J. Leon-Reyes A. Van der Ent S. Van Wees S. C. M. (2009). Networking by small-molecule hormones in plant immunity. *Nat. Chem. Biol.* 5 308–316. 10.1038/nchembio.164 19377457

[B79] Piubelli G. C. Hoffmann-Campo C. B. De Arruda I. C. Franchini J. C. Lara F. M. (2003). Flavonoid increase in soybean as a response to *Nezara viridula* injury and its effect on insect-feeding preference. *J. Chem. Ecol.* 29 1223–1233. 10.1023/a:1023889825129 12857032

[B80] Piubelli G. C. Hoffmann-Campo C. B. Moscardi F. Miyakubo S. H. Neves De Oliveira M. C. (2005). Are chemical compounds important for soybean resistance to *Anticarsia gemmatalis*? *J. Chem. Ecol.* 31 1509–1525. 10.1007/s10886-005-5794-z 16222789

[B81] Prochaska T. J. Donze-Reiner T. Marchi-Werle L. Palmer N. A. Hunt T. E. Sarath G. (2015). Transcriptional responses of tolerant and susceptible soybeans to soybean aphid (*Aphis glycines* Matsumura) herbivory. *Arthr. Plant Inte.* 9 347–359. 10.1007/s11829-015-9371-2

[B82] Quist T. M. Sokolchik I. Shi H. Joly R. J. Bressan R. A. Maggio A. (2009). HOS3, an ELO-like gene, inhibits effects of ABA and implicates a S-1-P/ceramide control system for abiotic stress responses in *Arabidopsis thaliana*. *Mol. Plant* 2 138–151. 10.1093/mp/ssn085 19529829 PMC2639740

[B83] Ragsdale D. W. McCornack B. P. Venette R. C. Potter B. D. Macrae I. V. Hodgson E. W. (2007). Economic threshold for soybean aphid (*Hemiptera : aphididae*). *J. Econ. Entomol.* 100 1258–1267. 10.1093/jee/100.4.1258 17849878

[B84] Ralston L. Subramanian S. Matsuno M. Yu O. (2005). Partial reconstruction of flavonoid and isoflavonoid biosynthesis in yeast using soybean type I and type II chalcone isomerases. *Plant Physiol.* 137 1375–1388. 10.1104/pp.104.054502 15778463 PMC1088328

[B85] Rao K. V. Chattopadhyay S. K. Reddy G. C. (1990). Flavonoids with mosquito larval toxicity. *J. Agr. Food Chem.* 38 1427–1430. 10.1021/jf00096a028

[B86] Robert-Seilaniantz A. Grant M. Jones J. D. (2011). Hormone crosstalk in plant disease and defense: more than just jasmonate-salicylate antagonism. *Annu. Rev. Phytopathol.* 49 317–343. 10.1146/annurev-phyto-073009-114447 21663438

[B87] Rodriguez P. A. Bos J. I. B. (2012). Toward understanding the role of aphid effectors in plant infestation. *Mol. Plant Microbe Interact.* 26 25–30. 10.1094/mpmi-05-12-0119-fi 23035915

[B88] Schmutz J. Cannon S. B. Schlueter J. Ma J. Mitros T. Nelson W. (2010). Genome sequence of the palaeopolyploid soybean. *Nature* 463 178–183. 10.1038/nature08670 20075913

[B89] Schopfer C. R. Kochs G. Lottspeich F. Ebel J. (1998). Molecular characterization and functional expression of dihydroxypterocarpan 6a-hydroxylase, an enzyme specific for pterocarpanoid phytoalexin biosynthesis in soybean (*Glycine max* L.). *FEBS Lett.* 432 182–186. 10.1016/S0014-5793(98)00866-7 9720921

[B90] Sharma H. C. Norris D. M. (1991). Chemical basis of resistance in soya bean to cabbage looper, *Trichoplusia* ni. *J. Sci. Food Agr.* 55 353–364. 10.1002/jsfa.2740550304

[B91] Smith C. M. Boyko E. V. (2007). The molecular bases of plant resistance and defense responses to aphid feeding: current status. *Entomol. Exp. Et Appl.* 122 1–16. 10.1111/j.1570-7458.2006.00503.x

[B92] Son G. H. Wan J. Kim H. J. Nguyen X. C. Chung W. S. Hong J. C. (2011). Ethylene-responsive element-binding factor 5, erf5, is involved in chitin-induced innate immunity response. *Mol. Plant Microbe Interact.* 25 48–60. 10.1094/mpmi-06-11-0165 21936663

[B93] Storey J. D. Taylor J. E. Siegmund D. (2004). Strong control, conservative point estimation and simultaneous conservative consistency of false discovery rates: a unified approach. *J. R. Stat. Soc. Ser. B* 66 187–205. 10.1111/j.1467-9868.2004.00439.x

[B94] Studham M. MacIntosh G. C. (2012). Phytohormone signaling pathway analysis method for comparing hormone responses in plant-pest interactions. *BMC Res. Notes* 5:392. 10.1186/1756-0500-5-392 22846705 PMC3460778

[B95] Studham M. E. (2010). *Transcriptional Defense Response Of The Soybean Plant, Glycines Max, In Response To Infestation By The Soybean Aphid, Aphis Glycines*. Ames: Iowa State University.

[B96] Studham M. E. MacIntosh G. C. (2013). Multiple phytohormone signals control the transcriptional response to soybean aphid infestation in susceptible and resistant soybean plants. *Mol. Plant Microbe Interact.* 26 116–129. 10.1094/MPMI-05-12-0124-FI 22992001

[B97] Suzuki H. Takahashi S. Watanabe R. Fukushima Y. Fujita N. Noguchi A. (2006). An isoflavone conjugate-hydrolyzing beta-glucosidase from the roots of soybean (glycine max) seedlings: purification, gene cloning, phylogenetics, and cellular localization. *J. Biol. Chem.* 281 30251–30259. 10.1074/jbc.M605726200 16891302

[B98] Thompson G. A. Goggin F. L. (2006). Transcriptomics and functional genomics of plant defence induction by phloem-feeding insects. *J. Exp. Bot.* 57 755–766. 10.1093/jxb/erj135 16495409

[B99] Tilmon K. J. Hodgson E. W. O’Neal M. E. Ragsdale D. W. (2011). Biology of the soybean aphid, *Aphis glycines* (*Hemiptera: aphididae*) in the united states. *J. Integr. Pest Manag.* 2 A1–A7. 10.1603/ipm10016

[B100] Tjallingii W. F. (2006). Salivary secretions by aphids interacting with proteins of phloem wound responses. *J. Exp. Bot.* 57 739–745. 10.1093/jxb/erj088 16467410

[B101] Tuteja J. Vodkin L. O. (2008). ). Structural features of the endogenous CHS silencing and target loci in the soybean genome. *Crop Sci.* 48 S49–S68. 10.2135/cropsci2007.10.0542tpg

[B102] Urbanczyk-Wochniak E. Sumner L. W. (2007). MedicCyc: a biochemical pathway database for *Medicago truncatula*. *Bioinformatics* 23 1418–1423. 10.1093/bioinformatics/btm040 17344243

[B103] Van Eck L. Davidson R. M. Wu S. Zhao B. Y. Botha A.-M. Leach J. E. (2014). The transcriptional network of WRKY53 in cereals links oxidative responses to biotic and abiotic stress inputs. *Funct. Genomic* 14 351–362. 10.1007/s10142-014-0374-3 24777609 PMC4059961

[B104] Van Eck L. Schultz T. Leach J. E. Scofield S. R. Peairs F. B. Botha A.-M. (2010). Virus-induced gene silencing of WRKY53 and an inducible phenylalanine ammonia-lyase in wheat reduces aphid resistance. *Plant Biotechnol. J.* 8 1023–1032. 10.1111/j.1467-7652.2010.00539.x 20561246

[B105] Walling L. L. (2008). Avoiding effective defenses: Strategies employed by phloem-feeding insects. *Plant Physiol.* 146 859–866. 10.1104/pp.107.113142 18316641 PMC2259051

[B106] Walling L. L. Thompson G. A. (2012). in *Behavioral and Molecular-Genetic Basis of Resistance against Phloem-Feeding Insects*, eds Thompson G. A. van Bel A. J. E. (Hoboken: Wiley-Blackwell), 328–351. 10.1002/9781118382806.ch16

[B107] Whiteman N. K. Groen S. C. Chevasco D. Bear A. Beckwith N. Gregory T. R. (2011). Mining the plant–herbivore interface with a leafmining *Drosophila* of *Arabidopsis*. *Mol. Ecol.* 20 995–1014. 10.1111/j.1365-294X.2010.04901.x 21073583 PMC3062943

[B108] Wille B. D. Hartman G. L. (2008). Evaluation of artificial diets for rearing *Aphis glycines* (*Hemiptera: Aphididae*). *J. Econ. Entomol.* 101 1228–1232. 10.1093/jee/101.4.1228 18767731

[B109] Wu Z. Irizarry R. A. Gentleman R. Martinez-Murillo F. Spencer F. A. (2004). Model-based background adjustment for oligonucleotide expression arrays. *J. Am. Stat. Assoc.* 99 909–917. 10.1198/016214504000000683

[B110] Yi J. Derynck M. R. Chen L. Dhaubhadel S. (2010). Differential expression of CHS7 and CHS8 genes in soybean. *Planta* 231 741–753. 10.1007/s00425-009-1079-z 20016991

[B111] Yu O. McGonigle B. (2005). Metabolic engineering of isoflavone biosynthesis. *Adv. Agro.* 86 147–190. 10.1016/S0065-2113(05)86003-1

[B112] Yun H. S. Kang B. G. Kwon C. (2016). *Arabidopsis* immune secretory pathways to powdery mildew fungi. *Plant Signal. Behav.* 11:e1226456. 10.1080/15592324.2016.1226456 27562527 PMC5257168

[B113] Zabala G. Zou J. Tuteja J. Gonzalez D. O. Clough S. J. Vodkin L. O. (2006). Transcriptome changes in the phenylpropanoid pathway of *Glycine Max* in response to *Pseudomonas syringae* infection. *BMC Plant Biol.* 6:26. 10.1186/1471-2229-6-26 17083738 PMC1636052

[B114] Zavala J. A. Mazza C. A. Dillon F. M. Chludil H. D. BallarÉ C. L. (2015). Soybean resistance to stink bugs (*Nezara viridula* and *Piezodorus guildinii*) increases with exposure to solar UV-B radiation and correlates with isoflavonoid content in pods under field conditions. *Plant Cell Environ.* 38 920–928. 10.1111/pce.12368 24811566

[B115] Zhang S. Zhang Z. Wen Z. Gu C. An Y.-Q. C. Bales C. (2017). Fine mapping of the soybean aphid-resistance genes rag6 and rag3c from glycine soja 85-32. *Theor. Appl. Genet.* 130 2601–2615. 10.1007/s00122-017-2979-0 28887657

[B116] Zhou Y.-Y. Luo S.-H. Yi T.-S. Li C.-H. Luo Q. Hua J. (2011). Secondary metabolites from *Glycine soja* and their growth inhibitory effect against *Spodoptera litura*. *J. Agr. Food Chem.* 59 6004–6010. 10.1021/jf200821p 21545185

[B117] Züst T. Agrawal A. A. (2016). Mechanisms and evolution of plant resistance to aphids. *Nature Plants* 2:15206. 10.1038/nplants.2015.206 27250753

